# Stable colony-stimulating factor 1 fusion protein treatment increases hematopoietic stem cell pool and enhances their mobilisation in mice

**DOI:** 10.1186/s13045-020-00997-w

**Published:** 2021-01-06

**Authors:** Simranpreet Kaur, Anuj Sehgal, Andy C. Wu, Susan M. Millard, Lena Batoon, Cheyenne J. Sandrock, Michelle Ferrari-Cestari, Jean-Pierre Levesque, David A. Hume, Liza J. Raggatt, Allison R. Pettit

**Affiliations:** Mater Research Institute-The University of Queensland, Faculty of Medicine, Translational Research Institute, 37 Kent St, Woolloongabba, 4102 Australia

**Keywords:** Colony-stimulating factor 1, Hematopoietic stem cells, Macrophages, HSC mobilisation

## Abstract

**Background:**

Prior chemotherapy and/or underlying morbidity commonly leads to poor mobilisation of hematopoietic stem cells (HSC) for transplantation in cancer patients. Increasing the number of available HSC prior to mobilisation is a potential strategy to overcome this deficiency. Resident bone marrow (BM) macrophages are essential for maintenance of niches that support HSC and enable engraftment in transplant recipients. Here we examined potential of donor treatment with modified recombinant colony-stimulating factor 1 (CSF1) to influence the HSC niche and expand the HSC pool for autologous transplantation.

**Methods:**

We administered an acute treatment regimen of CSF1 Fc fusion protein (CSF1-Fc, daily injection for 4 consecutive days) to naive C57Bl/6 mice. Treatment impacts on macrophage and HSC number, HSC function and overall hematopoiesis were assessed at both the predicted peak drug action and during post-treatment recovery. A serial treatment strategy using CSF1-Fc followed by granulocyte colony-stimulating factor (G-CSF) was used to interrogate HSC mobilisation impacts. Outcomes were assessed by in situ imaging and ex vivo standard and imaging flow cytometry with functional validation by colony formation and competitive transplantation assay.

**Results:**

CSF1-Fc treatment caused a transient expansion of monocyte-macrophage cells within BM and spleen at the expense of BM B lymphopoiesis and hematopoietic stem and progenitor cell (HSPC) homeostasis. During the recovery phase after cessation of CSF1-Fc treatment, normalisation of hematopoiesis was accompanied by an increase in the total available HSPC pool. Multiple approaches confirmed that CD48^−^CD150^+^ HSC do not express the CSF1 receptor, ruling out direct action of CSF1-Fc on these cells. In the spleen, increased HSC was associated with expression of the BM HSC niche macrophage marker CD169 in red pulp macrophages, suggesting elevated spleen engraftment with CD48^−^CD150^+^ HSC was secondary to CSF1-Fc macrophage impacts. Competitive transplant assays demonstrated that pre-treatment of donors with CSF1-Fc increased the number and reconstitution potential of HSPC in blood following a HSC mobilising regimen of G-CSF treatment.

**Conclusion:**

These results indicate that CSF1-Fc conditioning could represent a therapeutic strategy to overcome poor HSC mobilisation and subsequently improve HSC transplantation outcomes.

## Background

Mobilisation of hematopoietic stem and progenitor cells (HSPC) into peripheral blood is used to enable collection of enriched hematopoietic stem cells (HSC) for transplantation. This procedure is a successful approach used to treat a broad range of immune and hematopoietic malignancies and deficiencies. In up to 40% of patients referred for autologous transplant, insufficient numbers of HSPC are mobilised due to underlying morbidity or prior treatment impacts on the HSPC pool [1]. This deficiency can preclude HSC transplant in these poor mobilisers leaving no other effective treatment options [2]. Development of approaches to achieve HSC expansion prior to mobilisation would address this treatment gap [3].

HSC resides in specific locations called niches in the bone marrow (BM). BM resident macrophages are an integral component of HSC niches. Macrophage depletion in vivo is sufficient to drive mobilisation of HSC to blood [4–6] and prevent successful re-establishment of the HSC niche after total body irradiation [7]. Granulocyte colony-stimulating factor (G-CSF) was one of the first growth factors used to mobilise HSC [8] and remains the main compound to elicit HSPC mobilisation in donors and patients [9]. G-CSF triggers a complex array of mechanisms affecting HSC niche cellular and structural components [10], including direct effects on BM macrophages to elicit stem cell mobilisation [4, 11].

Colony-stimulating factor 1 (CSF1) (aka macrophage colony-stimulating factor, M-CSF) is required for the differentiation, survival and proliferation of tissue resident macrophages [12, 13]. Although clinical trial of CSF1 as an adjunct therapy for patients receiving HSC transplantation showed some benefit [14], it has not progressed into mainstream clinical care. Recombinant CSF1 is rapidly cleared by the kidneys and the clinical use of CSF1 required repeat high dose/continuous infusion (reviewed in [12]), making clinical use and pre-clinical studies impractical and/or cost prohibitive. To address CSF1 therapeutic limitations, Gow et al. engineered a pig CSF1 molecule conjugated to the Fc (CH-3) region of pig immunoglobulin IgG1a that greatly increased its circulating half-life [15]. Pig CSF1 is equally active on mouse and human macrophages [16], and short-term daily CSF1-Fc injection was well tolerated and effectively increased murine blood monocyte and tissue resident macrophage populations, including those in BM, mice [15], rats [17] and pigs [18]. AF647-labelled CSF1-Fc localised specifically to monocyte macrophages in tissues when injected into mice [19] confirming receptor specificity. However, the improved drug qualities of CSF1-Fc are associated with supraphysiologic pharmacokinetic properties [12, 15, 20]. This, as well as the resultant expansion of resident macrophages induced by treatment, could have complex consequences on hematopoiesis in BM and spleen that have not been investigated.

We hypothesised that CSF1-Fc treatment has direct and indirect impacts on the HSPC compartment that might be harnessed to improve HSC transplantation outcomes. Herein, we studied the temporal profile of the effects of an acute CSF1-Fc treatment regimen on resident macrophages, the HSPC pool and developing as well as mature leucocytes and erythroid cells. While the treatment initially reduced HSPC within the BM and triggered extramedullary hematopoiesis, the recovery phase following treatment was associated with an increase in total HSC that could be mobilised using subsequent G-CSF administration. We suggest that CSF1-Fc has potential utility in conditioning for more effective stem cell mobilisation.

## Methods

### Animals

All procedures complied with the Australian Code of Practice for the Care and Use of Animals for Scientific Purposes and were approved by The University of Queensland (UQ) Health Sciences Ethics Committee. C57BL/6 (CD45.2^+^) mice were sourced from Australian Resources Centre. Congenic B6.SJL-*Ptprc*^*a*^* Pep*^*c*^/BoyJ mice (B6.SJL CD45.1^+^), competitor transgenic red fluorescent protein (RFP) mice (generated by Professor Patrick Tam, Children’s Medical Research Institute, Sydney, Australia, and derived from TgN (ACTB-DSRed.T3) Nagy ES cells [21]), MacGreen mice containing *Csf1r*-enhanced green fluorescent protein (GFP) transgene [22] and C57BL/6-Tg(UBC-GFP)30^Scha/J^ GFP reporter mice were supplied from in-house breeding colonies. All mice used were 10-week-old females housed under specific pathogen-free conditions.

### In vivo studies

C57BL/6 mice were treated daily with either CSF1-Fc (1 mg/kg) or saline subcutaneously for 4 consecutive days as previously described [15]. Mice were killed at 7 and 14 days post-first saline or CSF1-Fc injection and peripheral blood [23], spleen [24], liver [25] and femoral bone [26] harvested for flow cytometry assessment, immunohistochemistry and colony-forming unit (CFU) assays. HSC mobilisation was performed 14 days post-first CSF1-Fc treatment by 3 consecutive bi-daily intraperitoneal injections of saline or G-CSF (125 µg/kg; Filgrastim, Amgen, Thousand Oaks, CA) with the same tissues collected the day following the last G-CSF injection*.*

### CFU assays

The CFU assays were performed as per Forristal et al. [27]. Briefly, the cytokine mix was prepared using optimal concentrations of recombinant mouse IL-3 (1000 U/ml), IL-6 (1000 U/ml), stem cell factor (SCF) (100 ng/ml) and granulocyte macrophage colony-stimulating factor (GM-CSF) in IMDM-supplemented media, and 60.9 µl of cytokine mix was added into 35 mm Petri dishes (Nullcon). In total, 2 µl of leukocyte suspension from 1 ml of sterile BM suspension from one femur, 10 µl of sterile 10 ml leukocyte suspension generated from half spleen and 10 µl of neat sterile blood were seeded on cytokine mix and covered with 1 ml of methyl cellulose. Colonies were counted after 7 days of culture incubated at 37ºC in 5% CO_2_.

### Competitive transplant assays

Competitive grafts were generated using 2 × 10^5^ BM from C57BL/6 donor mice treated with saline or CSF1-Fc (as per above) and equal numbers of BM cells from naïve UBC-GFP ‘competitor’ mice as described [28]. The BM cells were injected intravenously into lethally irradiated 10-week-old C57BL/6 recipient female mice. In another competitive transplant assay, C57BL/6 donor mice were treated with combination of CSF1-Fc or saline (as per above) plus G-CSF and whole heparinised blood was collected via cardiac puncture. In total, 20 µl of whole blood was then mixed with 2 × 10^5^ BM competitor cells from naïve RFP mice to generate the competitive graft [28]. The competitive grafts were intravenously injected into lethally irradiated 10-week-old B6.SJL CD45.1^+^ recipient mice. Blood was collected from recipients at 8-, 12- and 16-week post-transplant to quantify chimerism. Content in repopulation units (RU) was calculated for each individual recipient mouse according to CD45.2^+^ donor blood chimerism at 16-week post-transplantation using the following formula: $RU = \frac{{\left[ {D*C} \right]}}{{\left[ {100 - D} \right]}}$ where *D* is the percentage of donor CD45.2^+^ B and myeloid cells and C is the number of competing CD45.1^+^ BM RUs co-transplanted with the donor cells (*C* = 2 for 200,000 competing BM cells). RUs were then converted to per ml of blood [29]. Multilineage reconstitution was confirmed using a standard blood leucocyte lineage panel as previously described [30].

### Standard and imaging flow cytometry

Standard hematology analyser blood differential was used to enumerate blood cell counts. Myeloid lineage [7, 31], HSPC [7, 32], B cell subsets [33, 34] and red blood cell maturation [32, 35, 36] phenotyping were performed as detailed in Additional file 1: Table S1. Cell acquisition was performed on Beckman Coulter’s CyAn™ ADP Analyser (Beckman Coulter, USA), Cytoflex Analyser (Beckman Coulter, USA) or BD LSRFortessa™ X-20 (BD Biosciences, USA) for panels specified in Additional file 1: Table S1. Data analysis was performed using the FlowJo software (Tree Star Data Analysis Software, Ashland, OR).

For imaging flow cytometry BM was harvested and enriched for c-KIT^+^ (CD117) cells using magnetic activated cell sorting (MACS, Miltenyi Biotec) as previously described [37]. Enriched cells were stained using the antibody panel detailed in Additional file 1: Table S2 prior to image acquisition on a 3 laser AMNIS ImageStreamX MkII (Luminex Corporation) using the following settings: low flow rate, 40 × objective, 60 mW 405 nm laser power, 30 mW 488 nm laser power, 50 mW 642 nm laser power and the side scatter laser turned off. Data were compensated and analysed using IDEAS software (Life Science Research).

### Cell sorting, RNA isolation and quantitative PCR

BM was harvested and enriched for c-KIT + (CD117) cells using MACS as above. All cell sorting was performed using a MoFlo Astrios cell sorter (Beckman Coulter, USA), and HSPC subpopulations were gated as shown in Additional file 1: Fig. S5. Monocytes were sorted as F4/80^+^CD115^+^ cells as shown in Additional file 1: Fig. S1. Bone marrow-derived macrophages (BMM) were generated as previously described [38] and collected at day 7 of culture. Total RNA was extracted from sorted cells, BMM and total BM using the RNeasy Kit (Qiagen, UK) according to the manufacturer’s protocol. First-strand cDNA was synthesised using the iScript cDNA synthesis kit (Bio-Rad, USA). Quantitative PCR (qPCR) was performed using Maxima SYBR Green/ROX qPCR Master Mix (Thermo Scientific, USA) on a sequence detection system 7500 (Applied Biosystems, USA) and analysed by 7500 System SDS Software (version 1.3.1) using the following primers: m*Csf1r*-forward: 5′ CTGGGAGATCTTCTCGCTTGGT 3′; *mCsf1r*-reverse: 5′ CTCCAGGTCCCAGCAGGACT 3′. Relative expression to the housekeeping gene *Hprt* (*mHprt*-forward: 5′ GCCCCAAAATGGTTAAGGTTGC 3′; *mHprt* -reverse: 5′ AACAAAGTCTGGCCTGTATCCAAC 3′) was calculated using the power delta Ct method.

### Immunohistochemistry and immunofluorescence

For immunohistochemistry 5-µm sections of left hind limb and spleen, sampled from each block at 3 sectional depths 100 µm apart, were deparaffinized and rehydrated prior to staining with rat anti-F4/80 monoclonal antibody (Abcam, UK) or isotype control as previously described [39]. Sections were scanned at 40X magnification using Olympus VS120 slide scanner (Olympus, Japan) and analysed for percent area of chromogen staining in the entire section using Visiopharm VIS 2017.2 Image analysis software (Hørsholm, Denmark). Representative images were collected on an Olympus Bx50 microscope and cellSens standard software 7.1 (Olympus, Tokyo, Japan). For immunofluorescence, spleens were snap frozen in liquid nitrogen and embedded in Optimal Cutting Temperature (OCT; VWR, UK). Frozen sections (5 μm) were sequentially stained with anti-mouse F4/80-Alexa Fluor (AF) 647 (Abcam, UK), anti-mouse CD45R (B220)-AF488 (Biolegend, USA), anti-mouse CD169-AF594 (Biolegend, USA) or anti-mouse CD3ε-AF647 (Biolegend, USA). For Ki67 staining, paraffin tissues were dewaxed and retrieved using Diva Decloaker (Biocare Medical, USA) and sequentially stained with rabbit-anti mouse Ki67 (Abcam, UK) followed by species specific antibody coupled to AF594, followed with anti-mouse F4/80-AF647 (BD Bioscience, USA). All immunofluorescent images were counterstained with 4′,6-diamidino-2-phenylindole (DAPI; Thermo Fisher Scientific, USA). Immunofluorescence images were acquired on the Upright Motorized Olympus BX63 Upright Epifluorescence Microscope (Olympus Life Science, Australia). For morphometric analysis, digital microscopy images were analysed using ImageJ software (http://rsb.info.nih.gov/ij/) as previously described [40]. Briefly, all images were coded and assessed blindly at least 3 sectional depths. Background intensity thresholds were applied using an ImageJ macro which measures pixel intensity across all immunostained and non-stained areas of the images, and these were converted to percent staining area per mm^2^ of tissue [40].

### Statistical analysis

Details of all group/sample sizes and experimental repeats are provided in figure legends. Unless indicated otherwise, data are mean ± standard deviation (SD). Statistical analysis was performed using one-way ANOVA with Tukey’s multiple comparison test. In instances where there was evidence of non-normality identified by the Kolmogorov–Smirnov test, data were analysed by a Mann–Whitney U test. Values of *p* < 0.05 were accepted as significant.

## Results

### Acute CSF1-Fc treatment expands BM monocytes and resident macrophages.

**Fig. 1:**
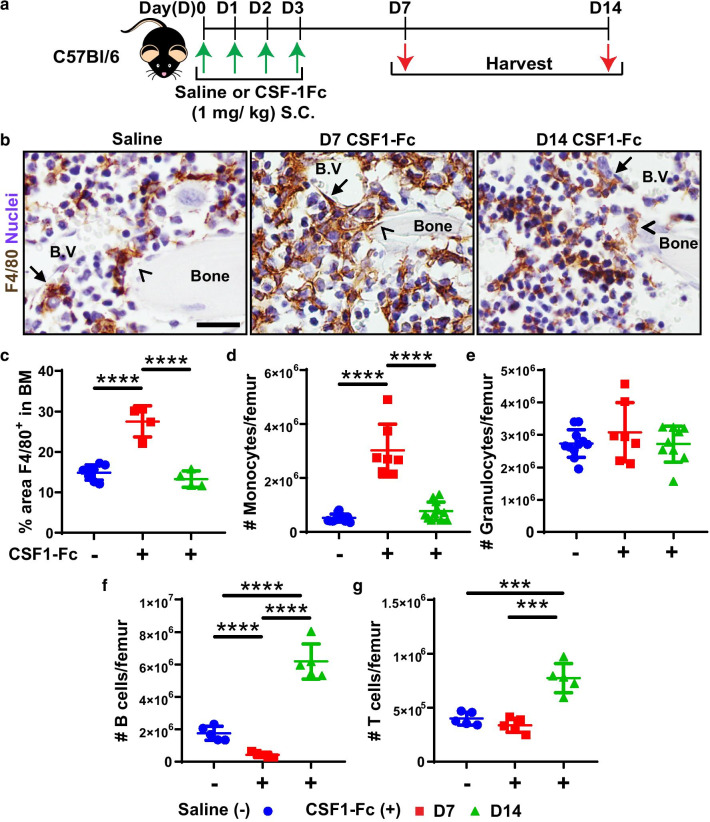
CSF1-Fc treatment induced significant expansion of BM resident macrophages. **a** Schematic of CSF1-Fc treatment regimen in C57BL/6 mice (D, day; S.C., subcutaneous). Tissues were harvested at 7 (D7) and 14 days (D14) post-first CSF1-Fc injection. **b** F4/80 immunohistochemistry (brown) in femoral BM sections of mice treated with saline (left panel) or CSF1-Fc at D7 (middle panel) and D14 (right panel). Closed arrows indicate perivascular macrophages, and arrowheads highlight endosteal macrophages. Sections were counterstained with hematoxylin (blue) and taken at 600X magnification. Scale bar = 20 µm. **c** Quantification of percent area of F4/80 staining in the femur of saline (blue circles, pooled D7 and D14 samples) and CSF1-Fc-treated mice at D7 (red squares) and D14 (green triangles) post-first injection. **d–g** Flow cytometry analysis to determine absolute number of cells per femur of **d** F4/80^+^Ly6G^neg^VCAM^neg^CD115^+^CD11b^+^ monocytes, **e** CD11b^+^Ly6G^+^ granulocytes, **f** CD11b^neg^CD3^neg^B220^+^ B cells and **g** CD11b^neg^B220^neg^CD3^+^ T cells in BM of saline controls or CSF1-Fc-treated mice. Flow cytometry representative raw data and gating provided in Additional file 1: Figs. S1 and S2. Each data point represents a separate mouse, and bars are mean ± SD. Statistical analysis was performed using one-way ANOVA Tukey’s multiple comparison test where ****p < 0.0001

CSF1-Fc treatment was previously demonstrated to increase F4/80^+^ and Gr-1^+^ BM cells at day 5, after 4 consecutive daily treatments [15]. However, sustained impacts and resolution kinetics of CSF1-Fc treatment on BM myeloid cells and broader impacts on other hematopoietic lineages were not mapped. Given CSF1-Fc remains substantially elevated in circulation for 72 h post-delivery, peak treatment effects could be delayed by at least 3 days post-treatment cessation. Consequently, CSF1-Fc was administered to female C57BL/6 mice daily for 4 days as described [15] and BM, spleen and blood were examined at 7 and 14 days post-initiation of treatment (Fig. 1a). F4/80^+^ resident BM macrophages and monocytes were present throughout the BM in saline-treated mice (Fig. 1b) including perivascular (closed arrows) and endosteal (open arrows) regions known to be enriched for HSC niches [41]. Seven days after initial CSF1-Fc treatment the frequency of F4/80^+^ cells and their ramification in BM was appreciably increased (Fig. 1b) and could be quantified as a 1.8-fold expansion in F4/80 staining area (Fig. 1c). This BM F4/80^+^ macrophage/monocyte expansion was transient and returned to baseline levels by day 14 (Fig. 1b, c). Flow cytometry confirmed an absolute increase in BM monocytes at day 7 which returned to baseline by day 14 (Fig. 1d; Additional file 1: Fig. S1a). There was no change in BM granulocyte number compared with saline at either time point (Fig. 1e; Additional file 1: Fig. S1b). BM B cell number was decreased at day 7 and rebounded to supraphysiologic levels at day 14 (Fig. 1f; Additional file 1: Fig. S2a). BM T cells were not immediately affected by treatment but were modestly increased at day 14 (Fig. 1g; Additional file 1: Fig. S2a). Blood changes mirrored BM observations as there was a transient 41% and 28% reduction of total white and red blood cell counts/ml at day 7 post-CSF1-Fc. Monocytosis was evident at day 7 but had resolved by day 14 (frequency relative to live CD45^+^ cells: saline 3.83 ± 0.98%; day 7 post-CSF1-Fc 10.3 ± 3.15%, *p* = 0.002; day 14 post-CSF1-Fc 4.5 ± 0.73%). Peripheral B cells were also transiently and significantly reduced at day 7 post-CSF1-Fc (frequency in live CD45^+^ cells: saline 42.81 ± 5.86%; day 7 post-CSF1-Fc 18.26 ± 2.78%; *p* < 0.000001) but rebounded to supraphysiologic frequency by day 14 compared to saline (52.4 ± 3.81%; *p* = 0.01). Peripheral T cells and granulocyte frequency were unchanged by CSF1-Fc treatment at the examined time points (not shown). Overall, CSF1-Fc treatment stimulated a robust but transient expansion of BM monocytes and macrophages, and while it disrupted B lymphopoiesis, this was transient and rapidly corrected by a rebound expansion of BM lymphocytes.

### Prolonged elevation of splenic resident macrophages post-CSF1-Fc treatment

**Fig. 2:**
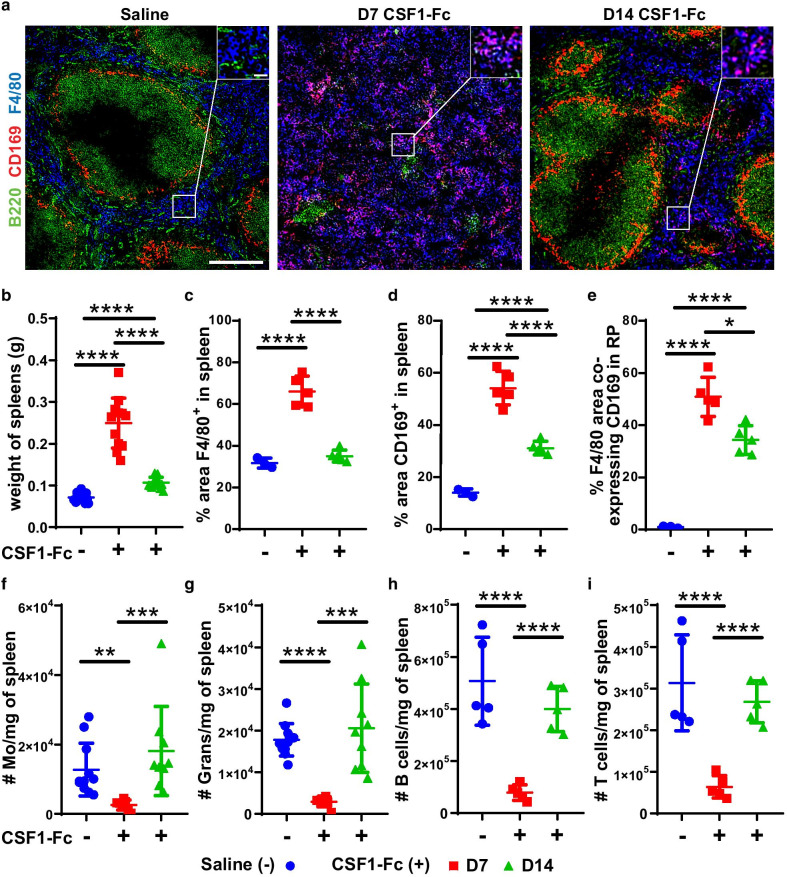
CSF1-Fc treatment transiently disrupts splenic architecture and altered red pulp macrophage phenotype. **a** Immunofluorescence labelling of F4/80 (blue), CD169 (red) and B220 (green) expression in spleen sections of mice treated with saline (left panel) or CSF1-Fc and assessed at D7 (middle panel) and D14 (right panel). Magnification = 100×; scale bar = 100 µm. Inset magnification = 600×; scale bar = 20 µm. **b** Spleen weights in saline controls (blue circles) or CSF1-Fc-treated mice at the D7 (red squares) and D14 (green triangles) time points. **c–e** Morphometric analysis of percent areas of **c** F4/80 immunolabelling, **d** CD169 immunolabelling and **e** F4/80 area co-labelled with CD169 per mm^2^ of tissue. **f–i** Flow cytometry analysis of total number of cells per mg of spleen of **f** F4/80^+^Ly6G^neg^VCAM^neg^CD115^+^CD11b^+^ monocytes (Mo), **g** CD11b^+^Ly6G^+^ granulocytes (Grans), **h** CD11b^neg^CD3^neg^B220^+^ B cells and **i** CD11b^neg^B220^neg^CD3^+^ T cells in saline control or CSF1-Fc-treated mice at both time points. Each data point represents a separate mouse, and bars are mean ± SD. Statistical analysis was performed using one-way ANOVA Tukey’s multiple comparison test where ****p < 0.0001, **p < 0.01 and *p < 0.05, *n* = 3 to 11 mice/group. Kolmogorov–Smirnov test revealed non-normality for data in graph **f**, therefore dictating use of a Mann–Whitney U test. Data for the saline control samples from D7 and D14 were pooled together in the graphical representations

Gow et al. previously reported an increase in spleen weight at day 5 after the same CSF1-Fc treatment regimen in transgenic MacGreen mice. This was associated with increased number of splenic GFP^+^ myeloid cells as well as increased area of the red pulp and marginal zone [15]. In situ assessment of the prolonged effects of CSF1-Fc treatment on splenic myeloid populations revealed overt disruption of gross splenic morphology at day 7 after CSF1-Fc treatment in non-transgenic mice (Fig. 2). There was dramatic decrease in both B220^+^ B cells (Fig. 2a) and CD3^+^ T cells (Additional file 1: Fig. S3a and c) with minimal identifiable white pulp on standard histology (not shown). There was also a complete loss of CD169^+^F4/80^low/−^ [42] marginal zone metallophilic macrophages (Fig. 2a) and a distinguishable marginal zone (Fig. 2a and Additional file 1: Fig. S3a). Disrupted splenic architecture was accompanied by sustained splenomegaly with spleen weight increased threefold 7 days after CSF1-Fc injection and remained 1.4-fold larger at 14 days post-initial treatment (Fig. 2b). Despite splenomegaly the number of Ki67^+^ proliferating cells present in spleen was substantially reduced at day 7 post-CSF1-Fc treatment (Additional file 1: Fig S3b and e). This contrasted to control and day 14 post-CSF1-Fc-treated spleens in which Ki67^+^ cells were common in both the red and white pulp (Additional file 1: Fig S3b and e). There was a substantial increase in F4/80^+^ macrophage-stained area within red pulp at day 7 post-treatment (Fig. 2a, c). In normal spleen, red pulp macrophages do not express CD169 [42, 43] and this was confirmed in saline-treated mice (Fig. 2a, e). Notably, at day 7 in CSF1-Fc-treated mice, the majority of red pulp macrophages in the expanded spleens of CSF1-Fc-treated mice acquired CD169 expression (Fig. 2a, d, e).

While the gross disorganisation of the spleen resolved by day 14, the altered F4/80^+^CD169^+^ red pulp macrophage phenotype persisted (Fig. 2a, d, e). Additionally, CD169^+^ macrophages were also detected within the CD3^+^ T cell zones of the white pulp in CSF1-Fc-treated mice in contrast to saline-treated controls (Additional file 1: Fig. S3a and d). Flow cytometry showed a significant decrease in absolute abundance of splenic monocytes (2.8-fold), granulocytes (3.3-fold), B cells (4.2-fold) and T cells (3.2-fold) at day 7 in CSF1-Fc-treated mice which resolved by day 14 (Fig. 2f–i). Overall, CSF1-Fc treatment temporarily disrupted splenic architecture, characterised by expansion of red pulp macrophages at the expense of other mature leukocytes, but induced a prolonged increase in spleen size, without overt in situ proliferation, accompanied by a persistent alteration in red pulp macrophage phenotype.

### CSF1-Fc treatment transiently increased HSPC populations in BM, spleen and liver.

CD169 is expressed by HSC [5, 7] and erythroid [44, 45] niche macrophages in the BM. Given both the sustained increase in spleen size and shift in splenic red pulp macrophage phenotype to express CD169 [5, 7], we assessed whether CSF1-Fc treatment induced extramedullary hematopoiesis and/or impacted HSPC populations in BM, spleen and liver at 7 and 14 days post-treatment. HSPC functional subsets were subdivided based upon signalling lymphocyte activation molecule (SLAM, CD150) marker [46] expression within lineage negative, c-KIT^+^ and Sca1^+^ cells (LSK): CD48^neg^CD150^+^ self-renewing HSC, CD48^neg^CD150^neg^ non-self-renewing multipotent progenitors (MPP) and CD48^+^ hematopoietic progenitor cells (HPC). Committed progenitor cells were gated as lineage negative, c-KIT^+^ and Sca1^neg^ cells (Additional file 1: Fig. S5). This strategy provides improved precision of long-term repopulating HSC segregation from other HSPC populations compared to a previously reported study of CSF1 actions on HSPC [47].

**Fig. 3:**
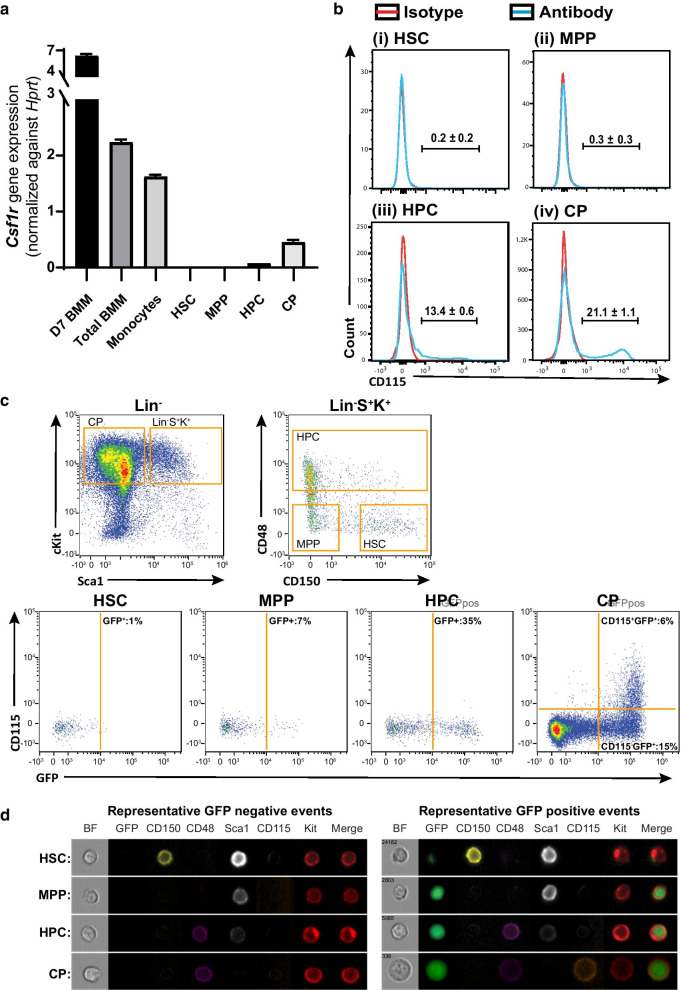
HSC does not express CSF1R. **a** Quantitative real-time PCR data demonstrating *Csf1r* mRNA expression in sorted HSC, MPP, HPC and committed progenitors as well as positive control samples of sorted monocytes, total BM and day 7 (D7) BMM. **b** Representative flow cytometry histograms of C57BL/6 mouse BM expression of CD115 (CSF1R) in (i) HSC, (ii) MPP, (iii) HPC and (iv) committed progenitors. Population gating strategies are exemplified in Additional file 1: Figs. S5 and S7. The histograms show antibody staining (blue lines) compared to appropriate isotype staining (red lines). **c** BM isolated from MacGreen mice was assessed by imaging flow cytometry with dot plots showing BM HSPC population gating strategy and subsequently CD115 and MacGreen GFP transgene expression in gated HSC, MPP, HPC and committed progenitors (CP). **d** Representative image panels showing individual bright field (BF) and specific antibody/transgene fluorophore images for representative cell events selected from either GFP-negative or GFP-positive gates in (**c**)

To reassess the potential for direct action of CSF1-Fc on HSC proposed by others [47], we analysed *Csf1r* mRNA and protein expression in SLAM marker gated HSPC populations (Fig. 3). Consistent with single cell transcriptional profiling indicating *Csf1r* mRNA was expressed predominantly in committed progenitors [48], *Csf1r* mRNA was undetectable in sorted HSC or MPP, but expression was low in HPC and committed progenitors (Fig. 3a). The relative amount of RNA was considerably greater in sorted monocytes, total BM or in vitro generated BM-derived macrophages (Fig. 3a) [49–51]. HSC and MPP did not express detectable CSF1R by standard flow cytometry, whereas it was detected on a small subpopulation of HPC and committed progenitors (Fig. 3b). Using imaging flow cytometry assessment of BM HSPC gated using the same LSK SLAM marker strategy isolated from MacGreen reporter mice [22], cell surface CSF1R protein (CD115) was undetectable in HSC, MPP and HPC (Fig. 3c, d), with the latter reflecting lower sensitivity of this technique versus standard flow cytometry. CD115 surface expression was detected on 6% of committed progenitors (Fig. 3c, d). Notably, 35% and 24% of HPC and committed progenitors, respectively, expressed the cytoplasmic MacGreen GFP reporter driven by the *Csf1r*-promoter (Fig. 3c, d), consistent with activation of *Csf1r* transcriptional machinery in these cells. Less than 1% of gated HSC events had detectable GFP expression (Fig. 3c), and when present, it was associated with debris artefact on the cell surface, not homogenous expression of GFP throughout the cytoplasm (Fig. 3d). In total, 7% of MPP expressed endogenous cytoplasmic GFP (Fig. 3c, d). Considering this with the lack of Csf1r mRNA expression (Fig. 3a), it suggests that a small number of MPP are transcriptionally primed to initiate *Csf1r* expression but that this has not yet progressed to native gene expression, or that it is below detectable levels of quantitative RT-PCR. These data confirm that HSC and MPP do not express CSF1R protein and therefore administered CSF1-Fc will not act directly on these cells.

**Fig. 4:**
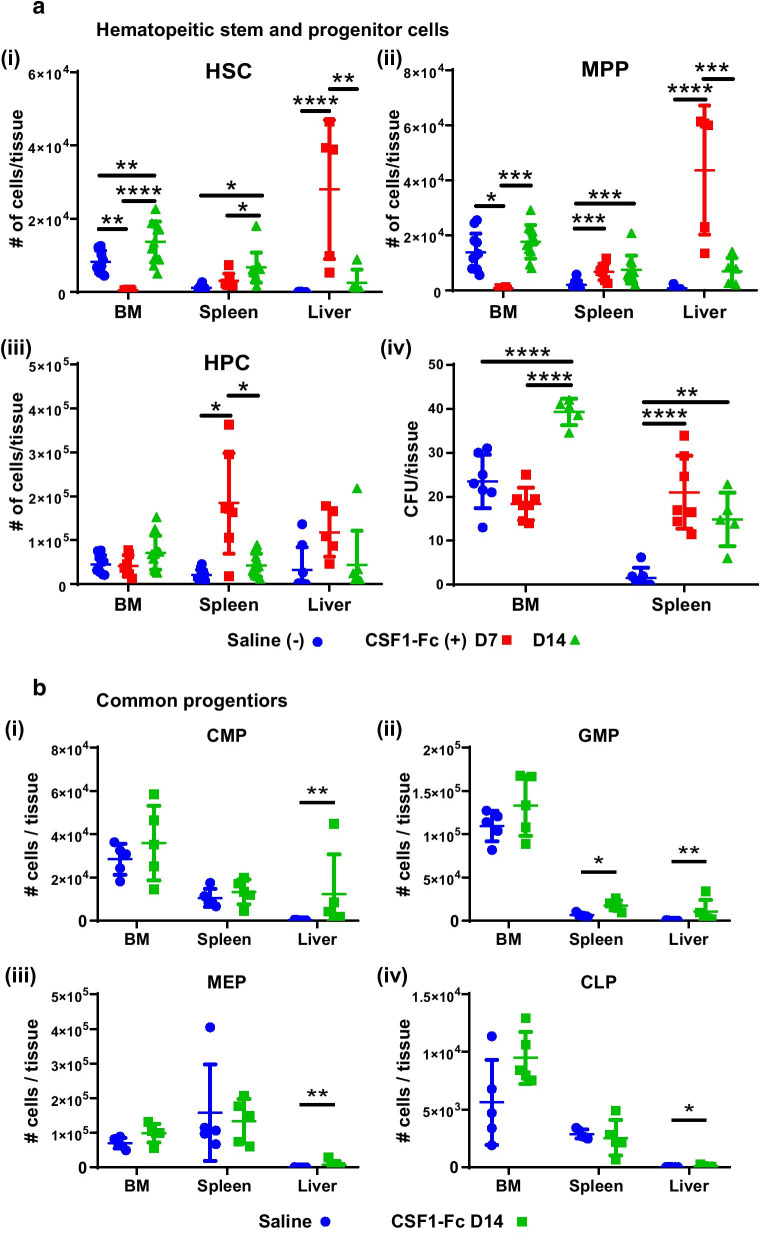
CSF1-Fc treatment is associated with a delayed increase in HSC and MPP in BM and spleen. **a** Flow cytometry analysis to determine the number of HSC (top left), MPP (top right) and HPC (bottom left) in BM, spleen or liver of C57BL/6 mice treated with saline (blue circles) or CSF1-Fc at D7 (red squares) or D14 (green triangles) post-first CSF1-Fc injection. Quantification of CFU in the BM (assay using single femur only) and spleen of saline or CSF1-Fc-treated mice at both time points (bottom right). **b** Number of CMP (top left), GMP (top right), MEP (bottom left) and CLP (bottom right) cells in BM, spleen and liver of C57BL/6 mice treated with saline (blue dots) or CSF1-Fc at D14 (green triangles) post-first CSF1-Fc injection. Population gating strategies are exemplified in Additional file 1: Fig. S7. Each data point represents a separate mouse, and bars are mean ± SD. Statistical analysis was performed using one-way ANOVA Tukey’s multiple comparison test where ****p < 0.0001, ***p < 0.0005, **p < 0.01 and *p < 0.05, n = 4 to 11 mice/group. Kolmogorov–Smirnov test revealed non-normality for liver data in graphs in (**a**), therefore dictating use of a Mann–Whitney U test. Data for the saline control samples from D7 and D14 time points were pooled together in the graphical representations

Nevertheless, CSF1-Fc treatment induced dynamic changes in organ distribution and number of phenotypic HSC and MPP. BM HSC and MPP were significantly decreased at 7 days post-treatment (Fig. 4a). MPP returned to normal (Fig. 4a), but BM HSC numbers rebounded to 1.7-fold above baseline at day 14 (Fig. 4a) post-CSF1-Fc. By contrast, BM HPC was unchanged by CSF1-Fc treatment (Fig. 4a). The CSF1-Fc-induced reduction of detectable BM phenotypic HSC and MPP at day 7 could be a consequence of downregulation of the key marker c-KIT in response to local environment changes triggered by CSF1-Fc [23, 52]. To confirm that number of HSC residing in BM was genuinely reduced by CSF1-Fc treatment, colony-forming unit (CFU; Fig. 4a) and competitive transplantation assays were performed (Additional file 1: Fig. S6a). There was no significant difference in CFU potential in BM from saline or day 7 post-CSF1-Fc-treated animals indicating HPC number and function was not impacted (Fig. 4a). However, competitive transplant confirmed loss of hematopoietic repopulation potential compared to saline-treated donor BM (Additional file 1: Fig. S6b) supporting actual decrease in long-term repopulating HSC resident in BM.

Unexpectedly, CSF1-Fc treatment induced a sustained increase of both phenotypic HSC and MPP in the spleen with these populations remaining elevated at day 14 (Fig. 4a). Splenic HPC was substantially increased at day 7 but returned to normal by day 14 (Fig. 4a). The treatment also led to a transient but striking increase in phenotypic HSC and MPP in the liver at 7 days post-treatment which resolved by day 14 (Fig. 4a), indicating liver had adapted to supported temporary lodgement of HSC, while BM and spleen were diverted to supporting elevated myelopoiesis (Figs. 1, 2). No change was noted in liver HPC number (Fig. 4a). Elevated HSPC mobilisation into blood was not evident at either time point examined after CSF1-Fc treatment (Additional file 1: Fig. S4). In the spleen, CFU activity aligned with the large and sustained increase in phenotypic HSPC driven by CSF1-Fc treatment (Fig. 4a). Overall, CSF1-Fc treatment produced a transient decrease in HSPC residence in BM at day 7 followed by a compensatory overshoot in the HSPC pool in BM and spleen at 14 days after initial treatment.

To determine whether the increased splenic HSC pool observed at 14 days post-CSF1-Fc treatment was an indirect consequence of compensatory extramedullary hematopoiesis, myeloid progenitors [7, 31] (Additional file 1: Fig. S7), lymphoid progenitors [32, 33, 53] (Additional file 1: Fig. S7), erythroblasts and reticulocytes [35, 36] were assessed in BM, spleen and liver at day 14 post-initial CSF1-Fc or saline treatment. No effect on common myeloid progenitor (CMP) number was observed at this time point in either BM or spleen, whereas they were increased in the liver (Fig. 4b). Granulocyte macrophage progenitor (GMP) number was not impacted in BM but was elevated in spleen and liver (Fig. 4b), and this is likely attributable to sustained CSF1-Fc treatment impacts on myeloid lineage dynamics. Megakaryocyte erythroblast progenitors (MEP, gating see Additional file 1: Fig. S7) and common lymphoid progenitors (CLP) were unchanged at day 14 in the BM and spleen but increased in the liver (Fig. 4b). These observations indicated extramedullary hematopoiesis is supported within the liver but not spleen at day 14 post-CSF1-Fc treatment.

The rebound in B cell populations noted in Figs. 1 and 2 in BM and spleen is likely attributable to significantly increased Pre-Pro B cells and Pre-B cell progenitor subsets in the BM (Additional file 1: Figs. S2 and S8a) at day 14 with no change in the spleen (not shown), where progenitor B cells do not reside under homeostatic conditions [34, 54]. All 3 transitional B cell populations that are normally located within spleen [33, 34] were increased at day 14 after CSF1-Fc treatment (Additional file 1: Figs. S2 and S8b) indicative of systemic elevation of B lymphopoiesis. Erythropoiesis, as indicated by number of proerythroblasts (Additional file 1: Fig. S8c) and erythroblasts (Additional file 1: Fig. S8d), was elevated in BM but not in the spleen when compared to saline control at day 14 post-CSF1-Fc. Reticulocytes number was equivalent to baseline in both BM and spleen (Additional file 1: Fig. S8e). Overall the data indicate that the increased splenic HSC pool was not an indirect consequence of extramedullary hematopoiesis during recovery from CSF1-Fc treatment. A more likely explanation consistent with induced expression of CD169 (Fig. 2a) is that the treatment has changed splenic capacity to support HSPC homing and/or temporary lodgement. On this basis, with appropriate timing, CSF1-Fc treatment has the potential to expand available phenotypic long-term repopulating HSC for mobilisation.

### G-CSF-induced HSPC mobilisation efficiency is enhanced by pre-treatment with CSF1-Fc

**Fig. 5:**
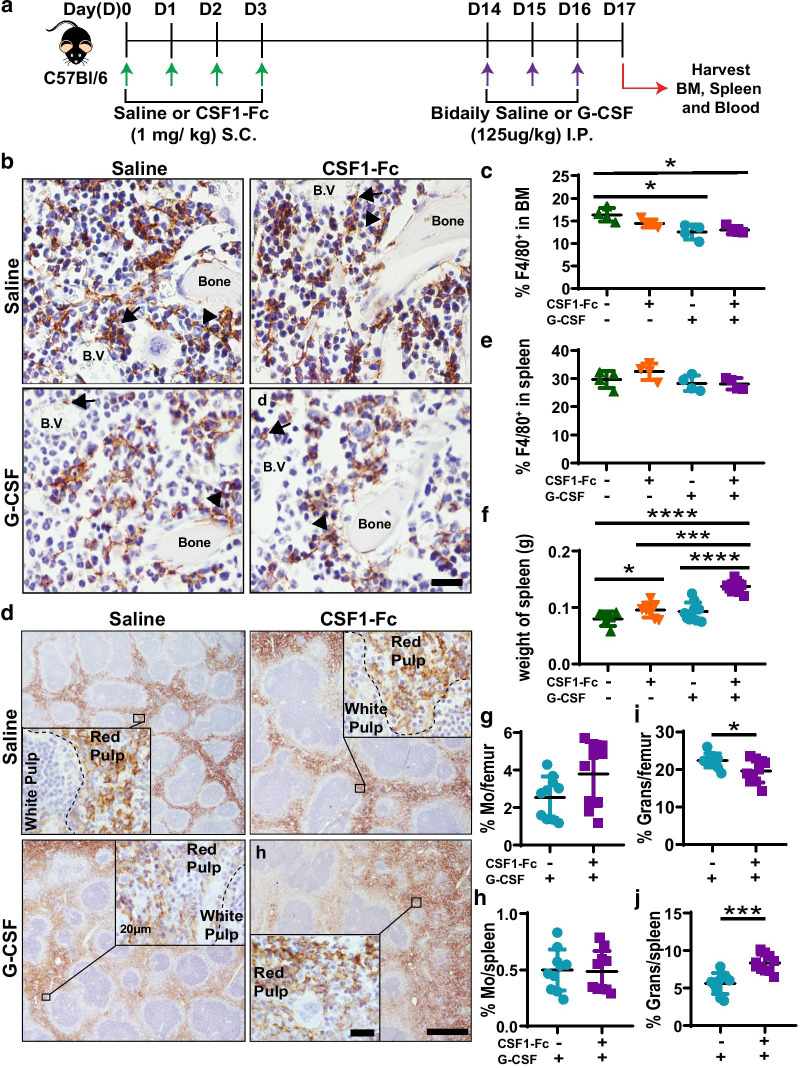
Tandem CSF1-Fc and G-CSF treatment had modest cumulative impacts on myeloid cells. **a** Schematic of tandem CSF1-Fc plus G-CSF treatment regimen administered to C57BL/6 non-transgenic mice. Briefly, mice were divided into 4 treatment groups: (1) once daily saline for 4 days followed by bi-daily saline treatment initiated 14 days later (saline + saline); (2) once daily CSF1-Fc for 4 days followed by bi-daily saline (CSF1-Fc + saline); (3) once daily saline for 4 days followed by bi-daily G-CSF treatment (saline + G-CSF); and (4) once daily CSF1-Fc for 4 days followed by bi-daily G-CSF treatment (CSF1-Fc + G-CSF). Tissues were harvested 17 days post-first CSF1-Fc injection. **b** Representative immunohistochemistry anti-F4/80 staining (brown) in femoral sections of mice treated as described above. F4/80^+^ BM resident macrophages (brown) are lining the endosteal (arrowheads) and perivascular (arrow) regions of the BM. Sections were counterstained with hematoxylin (blue) and taken at 600X magnification; scale bar = 20 µm. **c** Quantification of percent area of F4/80 staining in the femur of mice treated as above. **d** Representative immunohistochemistry anti-F4/80 staining (brown) in splenic sections of mice treated as above. Section were counterstained with hematoxylin (blue) and taken at 100X magnification; scale bar = 500 µm. Inset at 600X magnification; scale bar = 20 µm. **e** Quantification of percent area of F4/80 staining in the spleen of mice treated as above. **f** Weights of spleen of mice treated as above. **g–j** Flow cytometry analysis of the percentage of F4/80^+^Ly6G^neg^VCAM^+^CD115^+^CD11b^+^ monocytes (Mo) in BM (**g**) and spleen (**h**) or CD11b^+^Ly6G^+^ granulocytes (Grans) in BM (**i**) and spleen (**j**) of mice treated with either saline + G-CSF or CSF1-Fc + G-CSF. Each data point represents a separate mouse, and bars are mean ± SD. Statistical analysis was performed using one-way ANOVA Tukey’s multiple comparison test and unpaired Student's t test where ****p < 0.0001, ***p < 0.001, *p < 0.05

To test CSF1-Fc as a potential donor conditioning regimen, mice were treated with CSF1-Fc for 4 days followed by a HSPC mobilising regimen of G-CSF initiated at day 14 (CSF1-Fc + G-CSF, Fig. 5a). Consistent with a previous study [4], G-CSF treatment reduced F4/80^+^ macrophage frequency in BM (Fig. 5b, c) but had no significant impact on F4/80^+^ macrophage frequency in spleen (Fig. 5d, e). These responses were not altered by CSF1-Fc conditioning (Fig. 5b–e). Spleen weights of CSF1-Fc + G-CSF-treated mice were significantly higher when compared to the other 3 treatment groups (Fig. 5f). For simplicity, only G-CSF-treated groups as the key comparator groups are shown in subsequent figures. Saline only or CSF1-Fc only controls harvested at day 17 confirmed minimal change in data trends compared to day 14 CSF1-Fc treatment outcomes (Figs. 1, 2, 3, 4) with representative key examples provided in Additional file 1: Fig. S9. Pre-CSF1-Fc treatment did not change monocyte frequency in BM (Fig. 5g) or spleen (Fig. 5h) of G-CSF-treated mice. BM granulocyte frequency was significantly reduced (Fig. 5i), while splenic granulocyte frequency was significantly increased (Fig. 5j) by the combination therapy compared to G-CSF treatment alone.

**Fig. 6:**
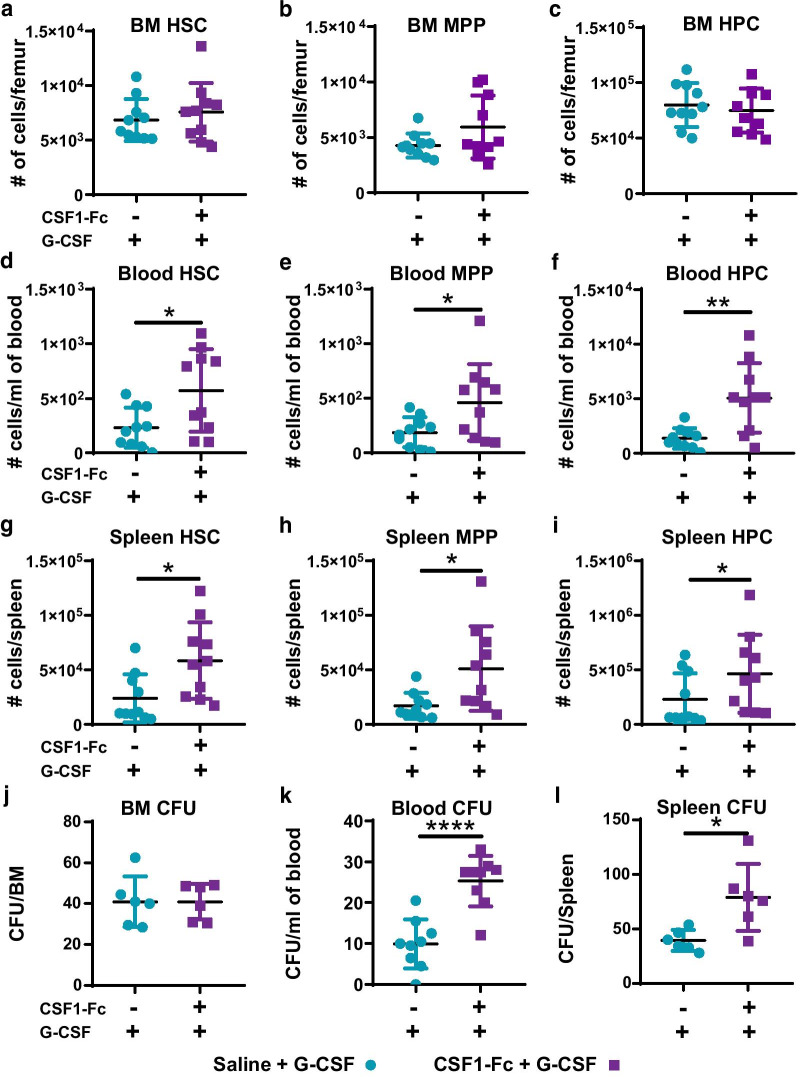
Sequential CSF1-Fc + G-CSF treatment mobilised HSPC more effectively than saline + G-CSF. Experimental schematic is described in Fig. 5(a). **a–i** Flow cytometry analysis to enumerate number of **a**, **d**, **g** HSC, **b**, **e**, **h** MPP and **c**, **f**, **i** HPC in BM (**a–c**), blood (**d–f**) and spleen (**g–i**) of mice treated as indicated. Population gating strategies are exemplified in Additional file 1: Fig. S5. Quantification of colony-forming units (CFU) in the **j** BM (assay using single femur only), **k** blood and **l** spleen in mice treated as indicated. Each data point represents a separate mouse, and bars are mean ± SD. Statistical analysis was performed using one-way ANOVA Tukey’s multiple comparison test where ****p < 0.0001, **p < 0.01 and *p < 0.05

No significant change in HSC, MPP or HPC number in BM of G-CSF mobilised mice was induced by pre-treatment with CSF1-Fc (Fig. 6a–c). However, the number of phenotypic HSC (2.5-fold), MPP (2.5-fold) and HPC (3.8-fold) mobilised by G-CSF was each increased in the blood of CSF1-Fc pre-treated mice compared to saline + G-CSF mice (Fig. 6d–f). Similar relative increases in HSC, MPP and HPC were detected in spleen in mice pre-treated with CSF1-Fc (Fig. 6g–i). The effect of the CSF1-Fc pre-treatment on progenitor cell mobilisation was confirmed by CFU assays using BM, blood or spleen (Fig. 6j–l).

**Fig. 7:**
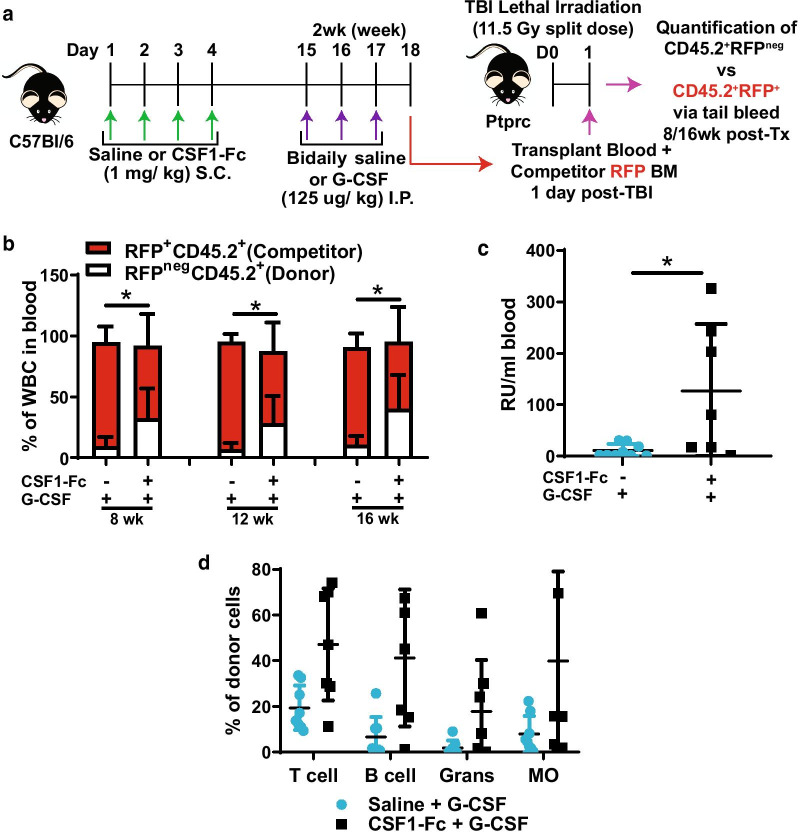
Combination of CSF1-Fc + G-CSF treatment improved the reconstitution potential of mobilised HSPC. **a** Schematic of competitive transplantation assay. Briefly, female donor C57BL/6 non-transgenic mice were treated with either once daily saline for 4 days followed by bi-daily G-CSF treatment (saline + G-CSF) 14 days later or once daily CSF1-Fc for 4 days followed by bi-daily G-CSF treatment (CSF1-Fc + saline) 14 days later as in Fig. 5a. At 17 days post-initial CSF1-Fc treatment blood was harvested from donor C57BL/6 mice from the two different treatment groups and then independently pooled with competitor BM from transgenic RFP mice and transplanted into lethally irradiated B6.SJL Ptprca recipients. Tail bleeds were performed at 8, 12 and 16 week post-transplantation to determine chimerism. **b** Quantification of blood chimerism of RFP^neg^CD45.2^+^ donors (white bars) and RFP^+^CD45.2^+^ competitors (red bars) in recipient mice that were transplanted with blood from saline + G-CSF or CSF1-Fc + G-CSF-treated donor mice. **c** Number of repopulating units (RU) per ml of blood transplanted in grafts collected from saline + G-CSF (light blue dots) and CSF1-Fc + G-CSF (black squares)-treated donors determined at 16 weeks post-competitive transplant. **d** Percent frequency of major mature cell lineages in peripheral blood contributed by test donor samples (saline + G-CSF or CSF1-Fc + G-CSF) in competitive transplant assay. Data are mean ± SD. Evidence of data distribution non-normality was identified by the Kolmogorov–Smirnov test, and statistical analysis was performed on data using by a Mann–Whitney U test where **p < 0.01 and *p < 0.05

The function of mobilised HSC from saline and CSF1-Fc pre-treated mice was assessed using a competitive transplantation assay (Fig. 7a). Recipients that received a mobilised blood graft from saline + G-CSF control donors had detectable but low frequency of donor-derived blood leukocytes over 16 weeks post-transplant (Fig. 7b), whereas the frequency of donor-derived blood leukocytes (Fig. 7b) was significantly increased when it was sourced from donors that had CSF1-Fc + G-CSF treatment. Calculation of repopulating units within the grafts showed a significant increase in the number of repopulating units/ml in the blood of mice mobilised with G-CSF +CSF1-Fc compared to saline + G-CSF (Fig. 7c). Pre-treatment with CSF1-Fc did not bias the multilineage reconstitution potential of G-CSF-mobilised HSC (Fig. 7d). These results confirm that prior treatment with CSF1-Fc improved G-CSF-induced stem cell mobilisation efficiency and reconstitution potential.

## Discussion

The current study characterised hematopoietic impacts of treatment with a modified CSF1-Fc molecule that has improved drug qualities [15]. The expansion of monocytes and macrophages in BM and spleen following CSF1-Fc treatment was even more marked at day 7 (3 days after the last treatment) than at day 5 as studied previously [15]. This is consistent with the prolonged half-life of CSF1-Fc. Importantly, treatment impacts were rapidly reversible, reminiscent of transient effects of either 14-day continuous [55] or daily [56] infusion of unmodified CSF1 in human clinical trials. At the time of peak response to CSF1-Fc, the prolonged excessive production of mature myeloid cells in BM and spleen occurred at the expense of normal hematopoiesis including disruption of BM HSC niche homeostasis. These impacts were again transient and largely resolved within a week of the peak treatment effect. We unexpectedly reveal that CSF1-Fc therapy caused a delayed increase in the total BM and spleen HSC pool, and we showed that this could be manipulated to achieve enhanced HSC mobilisation for transplantation.

Both CSF1 therapeutic potential and elucidation of CSF1-mediated in vivo biology have been hampered by the challenging practicalities of exogenous delivery of native CSF1. Continuous infusion of 150 µg/kg/day of native CSF1 in clinical trial resulted in only a transient increase in blood monocytes, peaking around day 7–8. Similarly, ongoing growth factor infusion [55] and repeat high dose regimens did not result in cumulative effects in animal models [57, 58]. Macrophages themselves clear CSF1 from the circulation by CSF1R-mediated endocytosis [59]. Consequently, the combination of treatment induced expansion of tissue macrophages, added to efficient renal clearance of native CSF1, likely culminate in rapid depletion of available growth factor from circulation despite ongoing treatment. Our and previous observations [15, 20] demonstrate that avoidance of renal clearance through use of modified CSF1-Fc is sufficient to achieve additive and sustained growth factor effects that parallel drug dose and predicted bioavailability. Further preclinical application of CSF1-Fc has potential to accelerate discovery regarding the usefulness of targeting the CSF1-CSF1R axis to modulate macrophages in clinical applications including organ regeneration [60], chemotherapy consolidation [55] and HSC transplantation [14, 61]. Delivery of a hematopoietic growth factor to patients with underlying cancer, particularly hematological malignancies, would need to proceed with caution. However, at least 10 clinical trials have been conducted using high dose CSF1 therapy in cancer patients, including melanoma, refractory solid tumours, lymphoma and leukaemia. No adverse impacts relating to accelerated cancer progression were reported (reviewed in [12]). Importantly, clinical trials using high dose CSF1 in leukaemias, including acute myeloid leukaemia, did not impact relapse rate [14, 56].

We provide compelling evidence that HSC does not express CSF1R and consequently the HSC treatment impacts are not due to direct action of CSF1-Fc. Furthermore, a next-generation knock-in *Csf1r* reporter model dependent on target translation confirmed CSF1R protein expression is restricted to the monocyte/macrophage lineage with expression initiating in Lin^−^Kit^+^Sca1^+^CD48^+^ multipotent progenitors [62]. Mossadegh-Keller et al*.* have previously suggested that HSC are directly responsive to CSF1 using a PU.1 reporter model [47]. However, it is now appreciated that the ‘HSC’ gating strategy used in this earlier study does not achieve precision segregation of HSPC subsets, with the reported data likely reflecting CSF1 action on CSF1R^+^ HPC within their gated population. Our observations that *Csf1r* RNA and CSF1R protein are undetectable in HSC aligns with a recently published single cell RNA sequencing study [48]. They are also consistent with *Spi1* knockout models which showed hematopoietic cells failed to express *Csf1r* in the absence of PU.1 [63, 64], and early studies indicating minimal proliferative effects on CFU-M in vitro [65]. It should also be noted that at the time HSC pool expansion was noted herein (i.e. 11 days after last CSF1-Fc injection), CSF1-Fc would have cleared from circulation.

In spleen, resident macrophages can contribute to retention of HSC during extramedullary myelopoiesis [66]. However, the observed delayed-CSF1-Fc-associated increase in splenic HSC was not accompanied by local extramedullary hematopoiesis, suggesting the lodged HSC were in a resting state. Acute CSF1-Fc treatment expanded mature F4/80^+^ resident macrophages within spleen at the expense of lymphocyte, granulocyte and even monocyte frequency/retention. The expansion was skewed toward maturation of a subset of F4/80^+^CD169^+^ red pulp macrophages that under physiologic conditions are present at very low frequency in spleen. This unusual F4/80^+^CD169^+^ red pulp macrophage phenotype persisted even after splenic morphology was reinstated, and macrophage frequency had returned to normal. F4/80^+^CD169^+^ macrophages support HSC in BM [5, 7]. We speculate that the phenotype shift in splenic macrophages reflects functional adaptation toward creating pseudo-BM niches for temporary maintenance of long-term repopulating HSC. This need for a temporary increase in HSC niche capacity BM may be in response to increased demand on the HSC pool, including the initial myelopoiesis and secondary lymphopoiesis demands that were triggered by CSF1-Fc treatment. Consequently, direct effects of CSF1-Fc on macrophages, combined with secondary effects on hematopoiesis homeostasis, converged to achieve the total HSC pool increase. Further studies are required to explicitly link the changed splenic macrophage phenotype with improved HSC-supportive function. Additional studies are also needed to confirm that CSF1-Fc treatment has not compromised HSC long-term repopulation integrity. Our data could be interpreted to conclude that CSF1-Fc has not compromised HSC quality. Disrupting BM HSC niche homeostasis does not automatically equate to compromise HSC quality, and our data show that the expression of c-KIT, which can be downregulated in response to stress [52], is maintained in both the HSC that rapidly relocated to liver and the expanded HSC pool within spleen. Additionally, the repopulation capacity was not compromised in competitive transplant, nor was there any impact on lineage potential of mobilised HSC after pre-treatment with CSF1-Fc. A possible interpretation of the rapid relocation of phenotypic long-term repopulating HSC to liver during the acute response to CSF1-Fc is that it represents a proactive protective measure due to CSF1-Fc-triggered alterations in BM microenvironment homeostasis. Future studies should include investigation of these possible outcomes.

We observed few proliferating cells in spleen at day 7 post-CSF1-Fc treatment, indicating that in situ proliferation of resident splenic macrophages was not a major mechanism of local expansion. Instead, the increase in splenic macrophage number is more likely due to influx of blood monocytes that subsequently differentiates into macrophages. These newly arrived monocytes undergo specific adaptation to the CSF1-Fc-altered splenic environment, which may be contributing to the shift in splenic macrophage phenotype. Gow et al. reported increased PCNA^+^ cells in spleen at day 5 post-CSF1-Fc treatment, so it is possible that the in situ macrophage proliferative response to CSF1-Fc had resolved by day 7 [15]. However, in this previous study the proliferating spleen cells were not confirmed to be macrophages and were predominantly located in the white pulp, which was not the dominant site of splenic macrophage expansion [15].

The potent monocytosis elicited by CSF1-Fc probably triggers known negative feedback loops directing compensatory reductions in lymphopoiesis and/or erythropoiesis [67]. We observed a significant impairment of BM B lymphopoiesis and temporary loss of lymphocytes in spleen. The observed reduction in BM B lymphopoiesis may be a secondary impact through altered osteoblast-lineage frequency or function, as per has been previously reported for suppressed B lymphopoiesis associated with mobilising regimens of G-CSF [68, 69]. Osteoblast function and frequency can be influenced by both osteoclasts and osteal macrophages [70]. CSF1-Fc treatment causes rapid expansion of bone-resorbing osteoclasts [15], and osteal macrophages are CSF1-responsive [26, 71], and paradoxically, systemic CSF1 treatment has an anabolic impact on bone [72]. Osteoblast-lineage cells in turn support B cell maturation [53]. Activation of this complex cellular feedback loop was not specifically examined in this study. The CSF1-Fc-induced increase in monocyte/macrophages could also result in supraphysiologic accumulation of growth factors and cytokines that are expressed by these cells, many of which have the capacity to influence hematopoiesis. For example, excessive monopoiesis could result in elevated secretion of interleukin-1 and/or interferons, which are known to trigger HSC proliferation [73]. Gene expression profiling of liver at day 5 post-CSF1-Fc treatment exposed increases in both pro- (*Il1, Il6* and *Tnf*) and anti-inflammatory (*Il10*) cytokines based on gene expression profiling [15]. Further investigation is required to understand the secondary indirect impacts of CSF1-Fc treatment.

A clinical challenge associated with autologous HSC transplantation is collection of sufficient HSC following mobilisation to achieve the required graft cell dose for successful transplant [1, 2]. The increase of total available HSC pool induced by CSF1-Fc treatment presented herein could address this unmet need. Enhanced mobilisation of HSC into blood of mice treated with CSF1-Fc + G-CSF therapy was accompanied by increased CFU activity in blood. Importantly increased reconstitution of all blood lineages in recipient mice was demonstrated using grafts from combination therapy versus G-CSF alone. As BM and spleen still contained HSC reserves after this mobilisation regimen, it is possible that HSC egress into blood could be further enhanced by treatment with a regimen that also included a CXCR4 antagonist [74, 75]. Clinical trials have explored the ability of granulocyte macrophage (GM)-CSF to enhance HSPC mobilisation in combination with G-CSF, including as a sequential regimen of GM-CSF prior to G-CSF. This combination therapy provided minimal or no advantage over G-CSF alone [76–79]. Therefore, expansion of the HSC pool is possibly a unique consequence of signalling through the CSF1R in myeloid lineage cells.

A remaining question is whether the long-term potential of the HSC within the CSF1-Fc + G-CSF donor graft is reduced due to CSF1-Fc exposure. Long-term serial transplant assays would be required to address this potential limitation. As discussed earlier, the collective data presented herein suggest that CSF1-Fc treatment induced 'stress' on the HSPC and committed progenitor compartment is contained and reversible. Mossadegh-Keller et al*.* similarly provided evidence that CSF1 treatment did not compromise long-term hematopoietic repopulating activity [47].

## Conclusion

This study has revealed the novel outcome of increased HSC pool as a consequence of CSF1-Fc therapy. This was due to HSC-indirect actions likely through increasing HSC niche supportive macrophages. Manipulation of this CSF1-Fc treatment outcome might be a viable strategy to correct HSC availability deficits in patients at risk of poor mobilisation outcomes.

## Supplementary information

**Additional file 1.** Figures showing gating strategies for cell populations of interest, companion data for the main figures and tables listing antibody details.

## Data Availability

All data generated or analysed during this study are included in this published article (and its Additional file 1). The raw datasets used and/or analysed during the current study are available from the corresponding author on reasonable requests.

## Abbreviations

BM: Bone marrow
CFU: Colony-forming units
CLP: Common lymphoid progenitor
CMP: Common myeloid progenitor
CP: Committed progenitor
CSF1-Fc: Colony-stimulating factor 1 Fc fusion protein
CSF1R: Colony-stimulating factor 1 receptor
G-CSF: Granulocyte colony-stimulating factor
GFP: Green fluorescent protein
GMP: Granulocyte-monocyte progenitor
HPC: Hematopoietic progenitor cells
HSC: Hematopoietic stem cells
HSPC: Hematopoietic stem and progenitor cells
LSK: Lineage negative, Sca1 positive, c-Kit (CD117) positive
MEP: Megakaryocyte-erythrocyte progenitor
MPP: Multipotent progenitor cells
