# Supplementary material for: Stable colony-stimulating factor 1 fusion protein treatment increases hematopoietic stem cell pool and enhances their mobilisation in mice
Source: J Hematol Oncol. 2021 Jan 6;14:3. doi: 10.1186/s13045-020-00997-w (PMC7786999; doi:10.1186/s13045-020-00997-w)
Supplement: Supplementary file 1 — Additional file 1. Figures showing gating strategies for cell populations of interest, companion data for the main figures and tables listing antibody details. [file 13045_2020_997_MOESM1_ESM.pdf]

Supplementary Information:

Stable colony stimulating factor 1 fusion protein treatment increases HSC pool and enhances their mobilisation in mice.

Simranpreet Kaur, Anuj Sehgal, Andy C. Wu, Susan M Millard, Lena Batoon, Cheyenne J. Sandrock, Michelle Ferrari-Cestari, Jean-Pierre Levesque, David A. Hume, Liza J. Raggatt and Allison R. Pettit.

Supp. Fig. 1. Myeloid lineage gating strategy in BM and spleen of saline or CSF1-Fc treated mice.

Representative flow cytometry plots of myeloid lineage in saline and CSF1-Fc treated mice at day (D)7 and D14 post-treatment. (a) BM monocytes were first gated as F4/80^{+}Ly6G^{neg} cells (orange gate) then further gated as VCAM-1^{neg}CD115^{+} cells (purple gate) that were predominantly CD11b^{+} (not shown). (b) BM granulocytes were identified as CD11b^{+}Ly6G^{+} cells (green gate). (c) Splenic monocytes were first gated as F4/80^{+}Ly6G^{neg} cells (orange gate) and then further gated as VCAM-1^{neg}CD115^{+} cells (purple gate) that were predominantly CD11b^{+} (not shown). (d) Splenic granulocytes were identified as CD11b^{+}Ly6G^{+} cells (green gate).

Supp. Fig. 2. Lymphocyte lineage gating strategy in BM and spleen of saline or CSF1-Fc treated mice.

Representative flow cytometry plots of B cell subsets and mature T cells in saline and CSF1-Fc treated mice at day (D)7 and D14 post-treatment in BM (a) and spleen (b). (a) In BM CD11b^{neg} cells were further gated as CD45R(B220)$^{+}$CD3^{neg} B cells (green gate) and CD45R(B220)^{neg}CD3^{+} T cells (red gate). Total CD45R(B220)$^{+}$ BM cells were analysed for

IgM and IgD analysis where Ig^{neg} B cells (orange gate) were further profiled for CD19 and CD43 expression to determine $CD19^{neg}CD43^{+}$ Pre-pro-B cells (blue gate), $CD19^{+}CD43^{+}$ Pro-B cells (green gate) and $CD19^{+}CD43^{neg}$ Pre-B cells (purple gate). (b) Spleens were analysed for the gated fractions $CD11b^{neg}CD3^{neg}$ cells (orange gate) and $CD11b^{neg}CD3^{+}$ T cells (red gate). $CD11b^{neg}CD3^{neg}$ cells were further profiled for total $CD4R(B220)^{+}$ B cells (green gate). The $CD4R(B220)^{+}CD93^{+}$ fraction (orange gate) were further analysed to identify transitional B cells: $IgM^{+}CD23^{neg}$ transitional-1 (T1) B cells (purple gate), $IgM^{+}CD23^{+}$ T2 B cells (green gate) and $IgM^{neg}CD23^{+}$ T3 B cells (blue gate).

Supp. Fig. 3. Immunofluorescence analysis of splenic architecture disruption and CD169^{+} cell frequency in white pulp 14 days post-CSF1-Fc treatment.

Immunofluorescence labelling of **(a)** CD169 (Red) and CD3 (Green) and (b) Ki67 (Red) and F4/80 (Green) in spleen sections of mice treated with saline (left panel) or CSF1-Fc at 7 days (D7; middle panel) and 14 days (D14; right panel) post-first injection. Magnification = 60X; scale bar = 500 µm. Inset magnification = 600X. **c-e)** Morphometric analysis of **(c)** percent area CD3 immunolabelling and **(d)** CD169 immunolabelling within T cell zones and **(e)** number of $Ki67^{+}$ cells in spleens of saline controls or CSF1-Fc treated mice at the D7 and D14 time points. Each data point represents a separate mouse and bars are mean ± SD. Evidence of data distribution non-normality was identified by the Kolmogorov–Smirnov test and statistical analysis was performed on data using by a Mann–Whitney U-test where ****$p < 0.0001$.

Supp. Fig. 4. HSPC were not mobilized to blood at day 7 or 14 post-CSF1-Fc treatment.

(a) Flow cytometry analysis to determine the number of **(a)** HSC, **(b)** MPP and **(c)** HPC in blood of C57BL/6 mice treated with saline (blue circles) or CSF1-Fc at D7 (red squares) or D14 (green triangles) post-first CSF1-Fc injection. Population gating strategies are exemplified

in Supp. Fig. 7. Each data point represents a separate mouse and bars are mean ± SD. Statistical analysis was performed using one-way ANOVA Tukey's multiple comparison test with no statistically significant difference observed between any group.

Supp. Fig. 5. HSPC gating strategy in BM, spleen and liver of saline or CSF1-Fc treated mice.

Representative flow cytometry analysis for committed progenitors and HSPC subsets in saline (top panel) or day (D) 7 (middle panel) and D14 (bottom panel) CSF1-Fc treated mice in (a) BM, (b) spleen and (c) liver. Committed progenitor cells were gated as lineage negative, c-Kit^{+} and Sca1^{neg} cells (black gate). HSPC were first gated as LSK (lineage negative, c-Kit^{+} and Sca1^{+} cells, orange gate). These were further fractioned as: CD48^{neg}CD150^{+} HSC (blue gate), CD48^{neg}CD150^{neg} MPP (green gate) and CD48^{+} HPC (purple gate).

Supp. Fig. 6. Competitive transplantation model and quantification of leukocyte chimerism in blood confirmed CSF1-Fc reduced BM HSC repopulation potential at 7 days after treatment.

(a) Schematic of competitive transplantation assay. Female donor C57BL/6 non-transgenic mice were treated with either daily saline or CSF1-Fc for 4 days. 2 x 10^{5}donor C57BL/6 BM cells of the two different treatment groups was collected 7 days post-first CSF1-Fc treatment and pooled with equal numbers of competitor UBG-GFP BM cells as described[27]. The BM cells were injected intravenously into lethally irradiated 10-week old C57BL/6 recipient female mice. (**b)** Tail bleeds were performed at 8-, 12- and 16-week post-transplantation and analysed for blood chimerism of CD45.2^{+}GFP^{neg} donors (white bars) and CD45.2^{+}GFP^{+} competitors (green bars) in recipient mice that receive BM from saline or CSF1-Fc treated donor mice. Data are mean ± SD. Statistical analysis was performed using one-way ANOVA Tukey's multiple comparison test where *$p<0.05$, n = 8 to 10 mice/group.

Supp. Fig. 7. Myeloid and lymphoid progenitor cell gating strategy in BM, spleen and liver of saline or CSF1-Fc treated mice.

Representative flow cytometry analysis for myeloid progenitor cells in (a) BM (b) spleen and (c) liver and lymphoid progenitor cells in (d) BM, (e) spleen and (f) liver in saline or day (D) 14 CSF1-Fc treated mice. (a-c) For myeloid progenitor cells, Lineage^{neg}Sca-1^{neg}cKit^{+} cells (orange gate) were gated into CD16/CD32^{int}CD34^{+} common myeloid progenitors (CMP; blue gate), CD16/CD32^{+}CD34^{+} granulocyte-macrophage progenitors (GMP; purple gate) and CD16/CD32^{neg}CD34^{neg} megakaryocyte erythroid progenitors (MEP; green gate). (d-f) For lymphoid progenitor cells, Lineage^{neg}IL-7rα^{+} (orange gate) were gated into Sca-1^{int}c-Kit^{int} cells (purple gate) and the expression of Flk2 (receptor for Flt3 ligand) was then analysed on this population of cells to determine numbers of common lymphoid progenitors (CLP; green gate).

Supp. Fig. 8. CSF1-Fc treatment prolonged recovery was not associated with splenic extramedullary haematopoiesis.

(a) Flow cytometry analysis of BM B cell progenitor subsets at 3 developmental stages: Pre-Pro B cells, Pro-B cells and Pre-B cells in BM of C57BL/6 mice treated as above. Population gating strategies are exemplified in Supp Fig. 2. **(b)** Flow cytometry analysis of splenic transitional (T1-3) B cell maturation: CD93^{+}CD23^{neg}IgM^{+} T1 B cells, CD93^{+}CD23^{+}IgM^{+} T2 B cells and CD93^{+}CD23^{+}IgM^{neg} T3 B cells. Population gating strategies are exemplified in Supp. Fig 2. **(c-e)** Flow cytometry analysis of **(c)** Ho^{+}Ter119^{low}CD71^{+} pro-erythroblasts, **(d)** Ho^{+}Ter119^{+}CD71^{+} erythroblasts and **(e)** Ho^{neg}Ter119^{+} reticulocytes BM (quantification in single femur only) and spleen of C57BL/6 mice treated as above. Each data point represents a separate mouse and bars are mean ± SD. Statistical analysis was performed using one-way

ANOVA Tukey's multiple comparison test where ****p<0.0001, ***p<0.0005, **p<0.01 and *p<0.05.

Supp. Fig. 9. CSF1-Fc effects were stable between day 14 and 17 post treatment initiation. Experiment is described in Fig. 5(a). Saline + saline (blue circles) and CSF1-Fc + saline (orange squares) control group treatment impact exemplars. Flow cytometry analysis to determine frequency of monocytes (MO) in BM **(a)** and spleen **(b)**. Enumeration of number of cells in BM, spleen or blood for **(c)** HSC, **(d)** MPP and **(e)** HPC of mice treated as indicated. Population gating strategies are exemplified in Supp Fig. 4. Statistical analysis was performed using one-way ANOVA Tukey's multiple comparison test where **p<0.01 and *p<0.05.

Supp. Fig. 10. Representative flow cytometry plots of competitive transplantation assay. Representative flow cytometry analysis for either RFP negative (black gate) or RFP positive (red gate) $CD45.2^+$ donor cells described in Figure 7a. Cells were pre-gated on live/total $CD45^+$ cells.

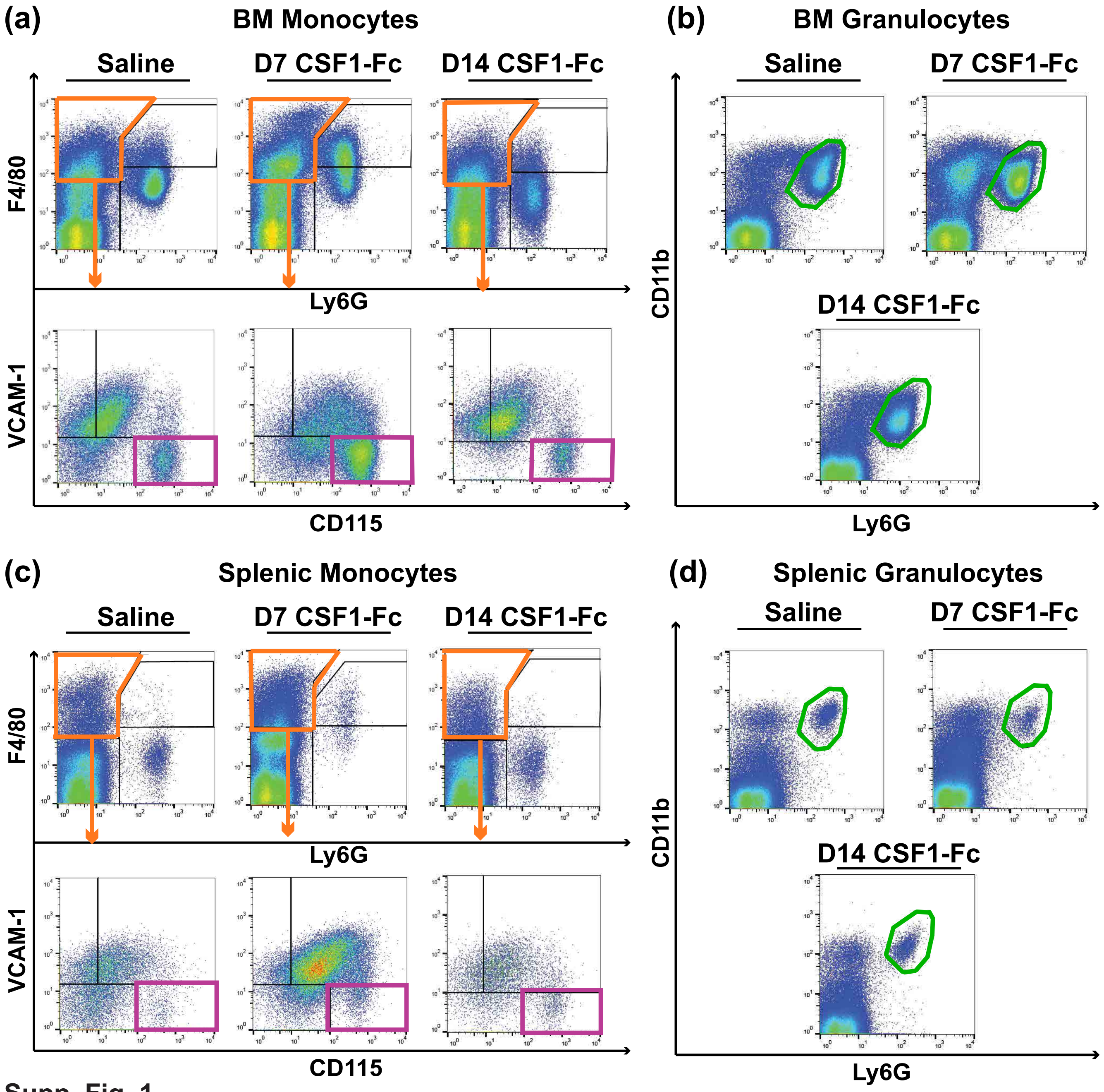

(a)
BM Monocytes
Saline
D7 CSF1-Fc
D14 CSF1-Fc
F4/80
Ly6G
VCAM-1
CD115
(b)
BM Granulocytes
Saline
D7 CSF1-Fc
D14 CSF1-Fc
CD11b
Ly6G
(c)
Splenic Monocytes
Saline
D7 CSF1-Fc
D14 CSF1-Fc
F4/80
Ly6G
VCAM-1
CD115
(d)
Splenic Granulocytes
Saline
D7 CSF1-Fc
D14 CSF1-Fc
CD11b
Ly6G

Supp. Fig. 1

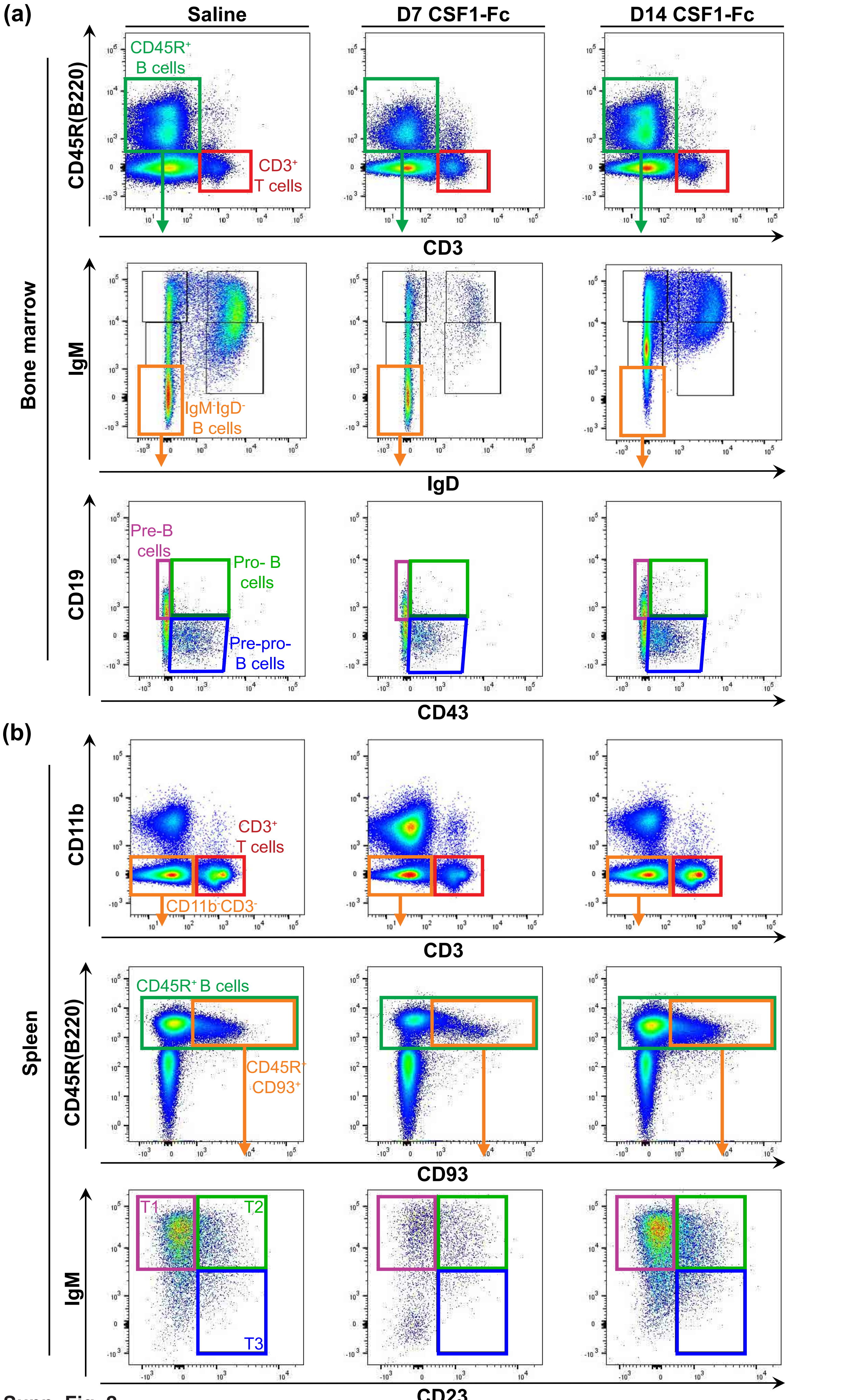
(a)
Saline
D7 CSF1-Fc
D14 CSF1-Fc
Bone marrow
CD45R(B220)
CD45R+ B cells
CD3+ T cells
CD3
IgM
IgM-IgD- B cells
IgD
CD19
Pre-B cells
Pro- B cells
Pre-pro- B cells
CD43
(b)
Spleen
CD11b
CD3+ T cells
CD11b-CD3-
CD3
CD45R(B220)
CD45R+ B cells
CD45R+ CD93+
CD93
IgM
T1
T2
T3
CD23

Supp. Fig. 2

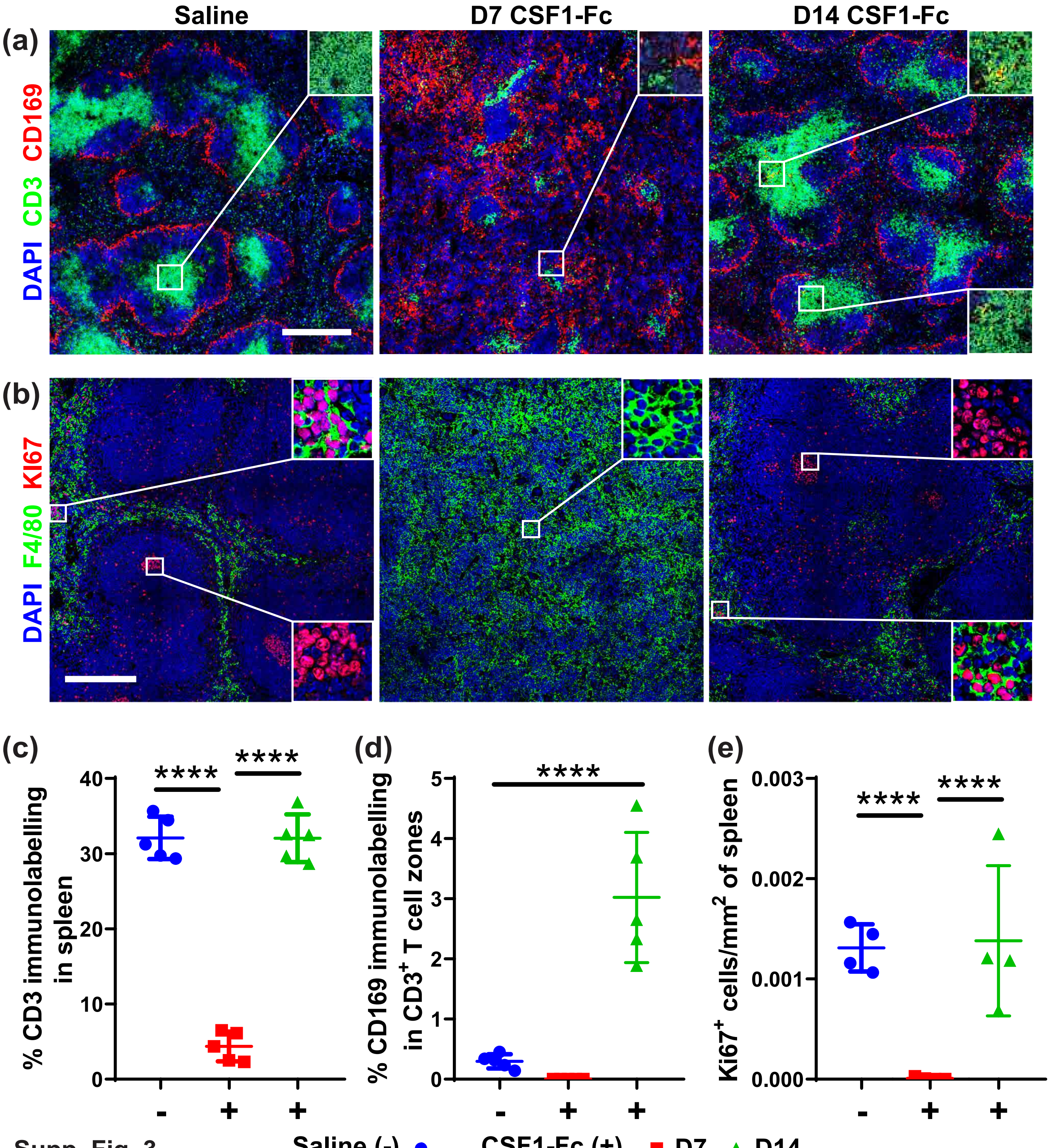
Saline
D7 CSF1-Fc
D14 CSF1-Fc
(a)
DAPI CD3 CD169
(b)
DAPI F4/80 KI67
(c)
% CD3 immunolabelling in spleen
(d)
% CD169 immunolabelling in CD3+ T cell zones
(e)
Ki67+ cells/mm2 of spleen
****
Saline (-)
CSF1-Fc (+)
D7
D14

Supp. Fig. 3

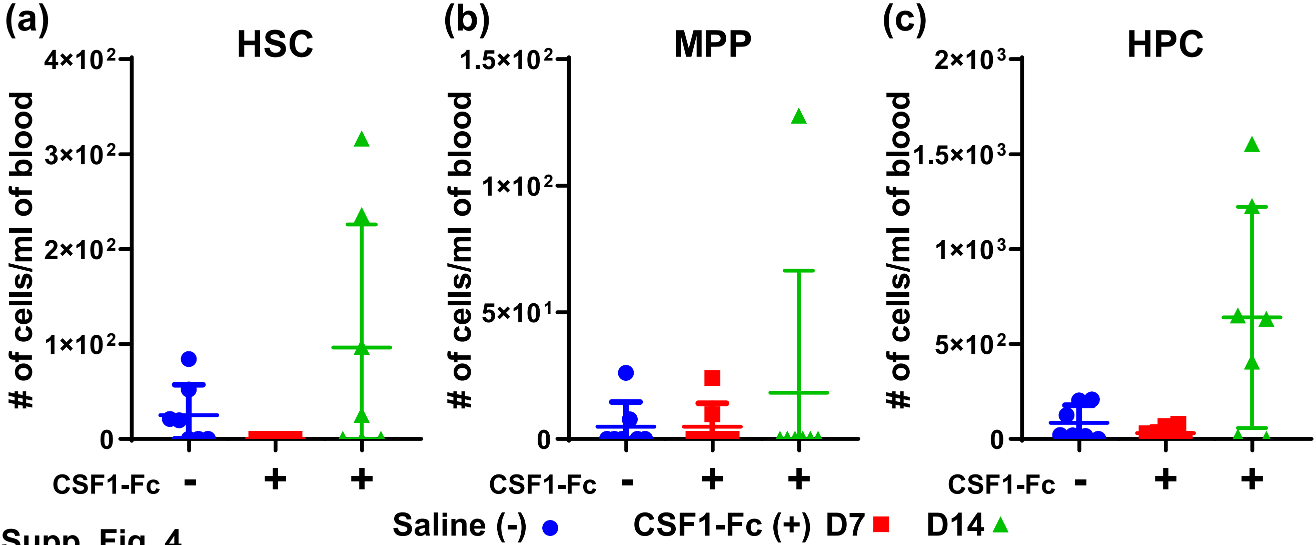
(a)
HSC
# of cells/ml of blood
4×10²
3×10²
2×10²
1×10²
0
CSF1-Fc - + +
(b)
MPP
1.5×10²
1×10²
5×10¹
0
CSF1-Fc - + +
(c)
HPC
2×10³
1.5×10³
1×10³
5×10²
0
CSF1-Fc - + +
Saline (-)
CSF1-Fc (+) D7
D14

Supp. Fig. 4

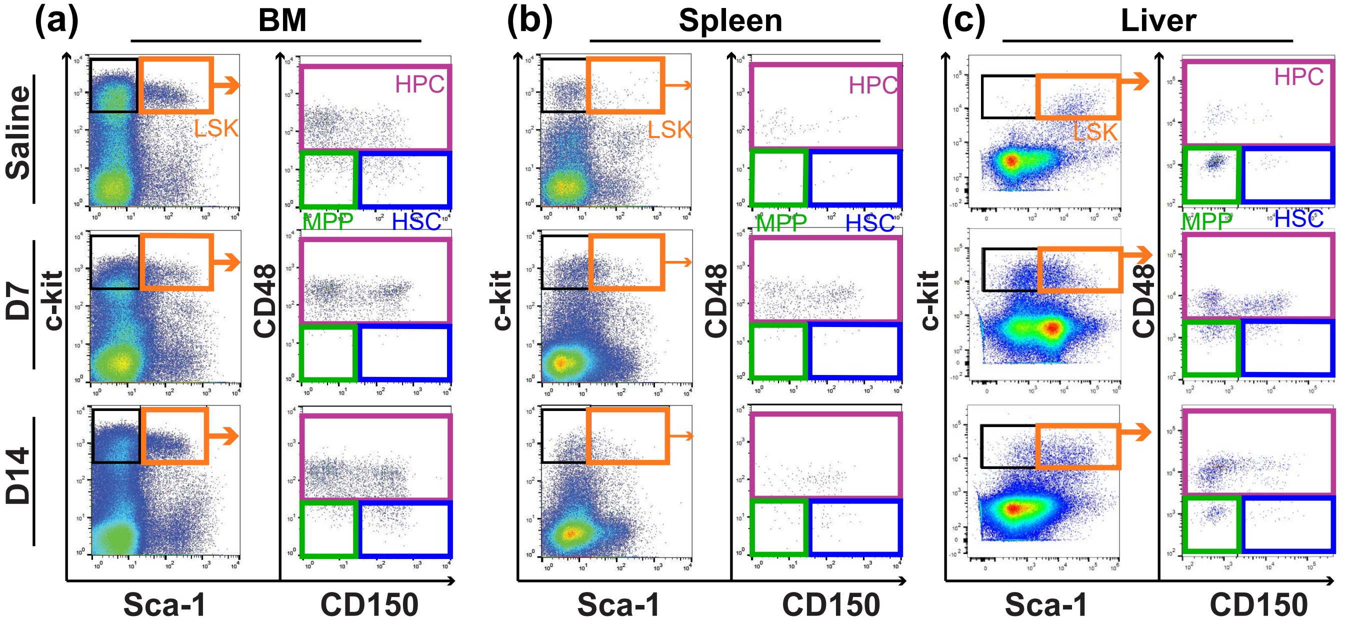
(a)
BM
(b)
Spleen
(c)
Liver
Saline
D7
D14
c-kit
CD48
LSK
HPC
MPP
HSC
Sca-1
CD150

Supp. Fig. 5

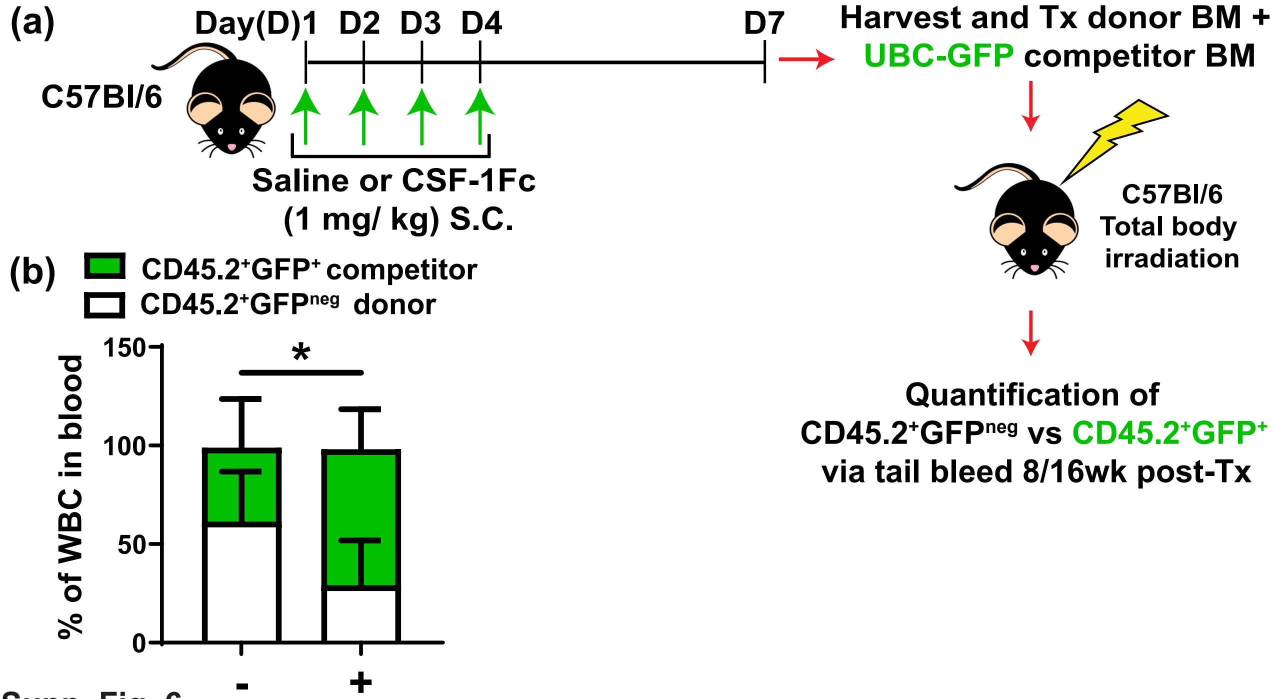
(a)
Day(D)1
D2
D3
D4
D7
C57Bl/6
Saline or CSF-1Fc
(1 mg/ kg) S.C.
Harvest and Tx donor BM +
UBC-GFP competitor BM
C57Bl/6
Total body
irradiation
Quantification of
CD45.2+GFPneg vs CD45.2+GFP+
via tail bleed 8/16wk post-Tx
(b)
CD45.2+GFP+ competitor
CD45.2+GFPneg donor
% of WBC in blood
150
100
50
0
*
-
+

Supp. Fig. 6

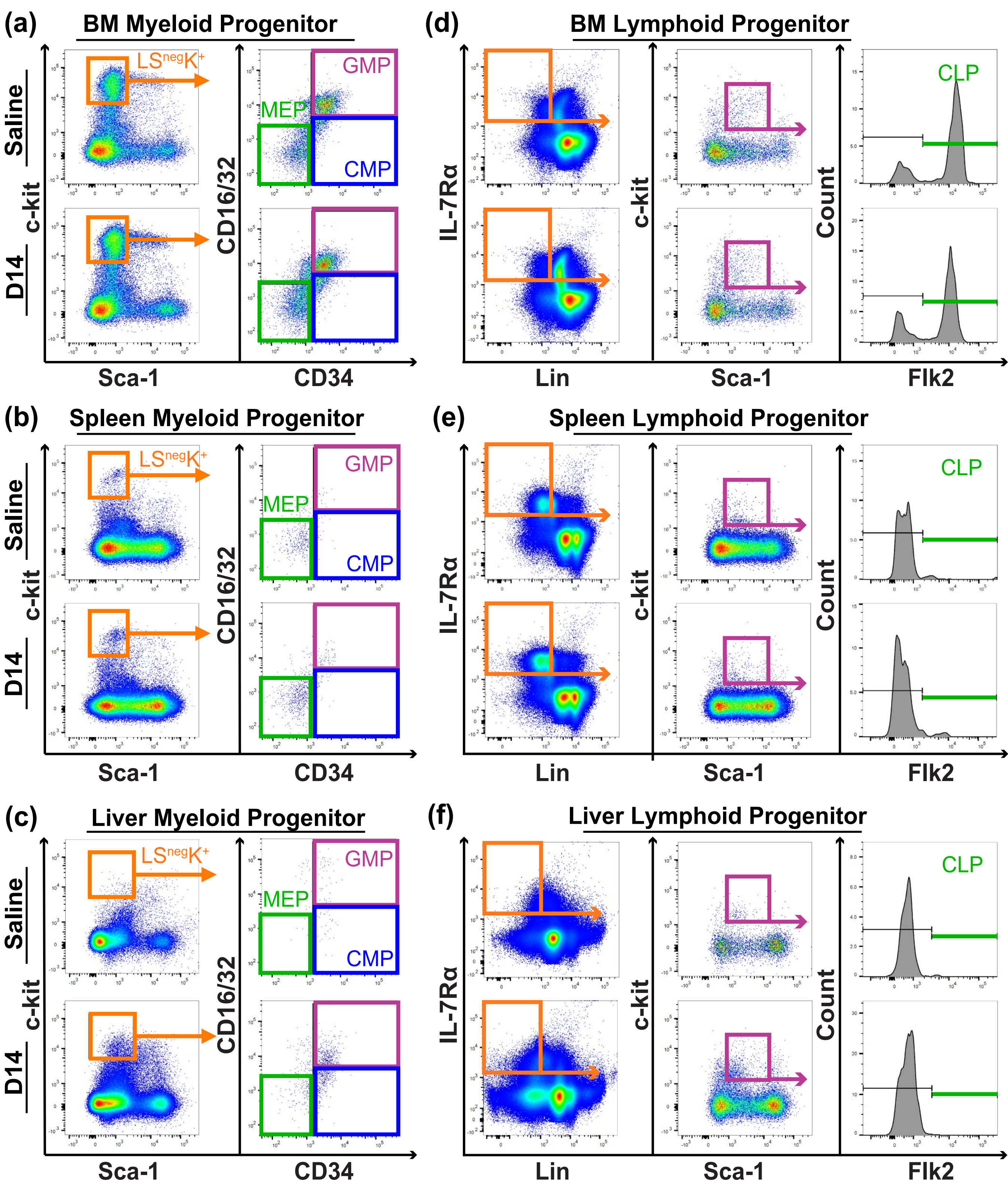
(a)
BM Myeloid Progenitor
(b)
Spleen Myeloid Progenitor
(c)
Liver Myeloid Progenitor
(d)
BM Lymphoid Progenitor
(e)
Spleen Lymphoid Progenitor
(f)
Liver Lymphoid Progenitor
Saline
D14
c-kit
Sca-1
LSnegK+
CD16/32
CD34
GMP
MEP
CMP
IL-7Rα
Lin
Count
Flk2
CLP

Supp. Fig. 7

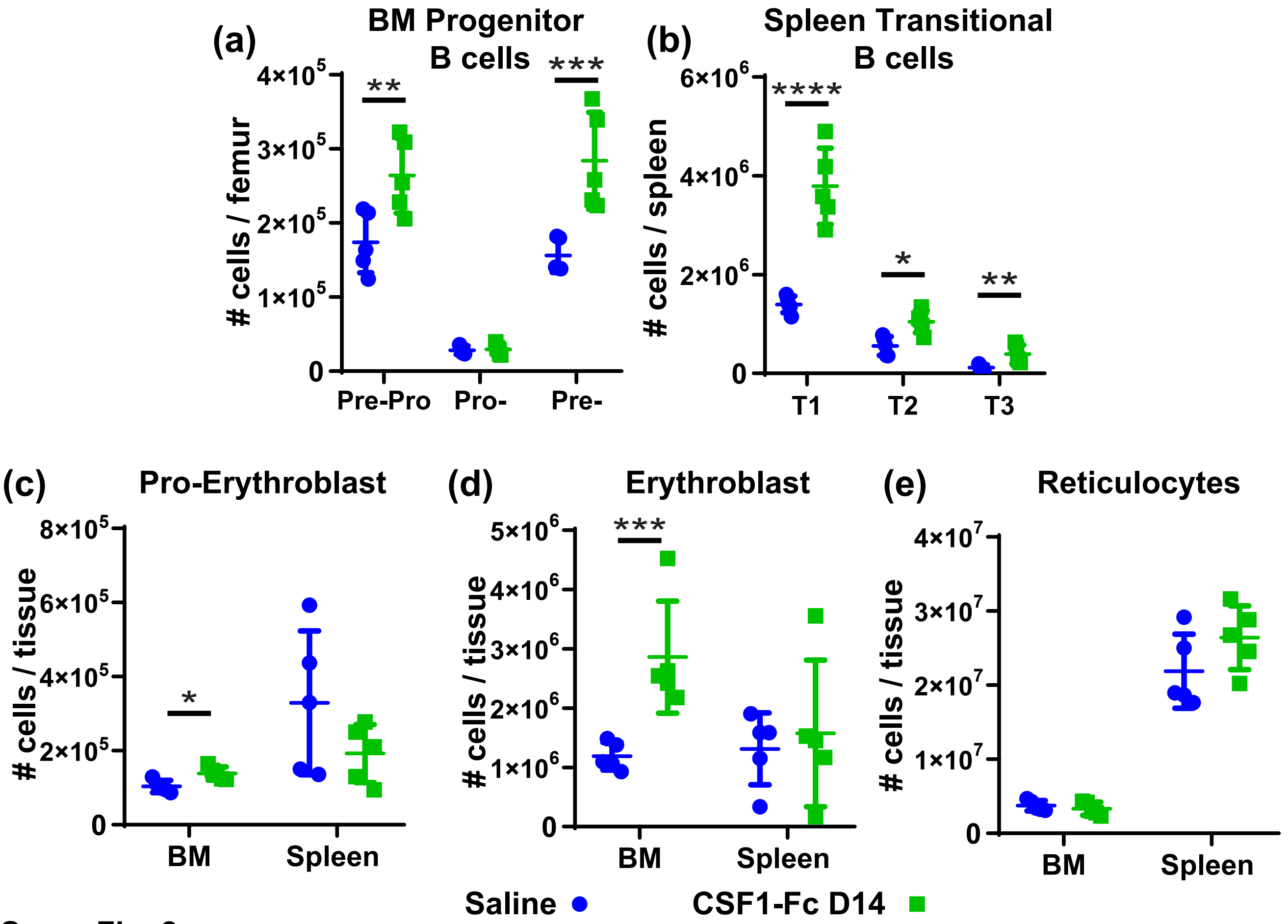
(a)
BM Progenitor B cells
cells / femur
4×10^5
3×10^5
2×10^5
1×10^5
0
**
***
Pre-Pro
Pro-
Pre-
(b)
Spleen Transitional B cells
cells / spleen
6×10^6
4×10^6
2×10^6
0
****
*
**
T1
T2
T3
(c)
Pro-Erythroblast
cells / tissue
8×10^5
6×10^5
4×10^5
2×10^5
0
*
BM
Spleen
(d)
Erythroblast
cells / tissue
5×10^6
4×10^6
3×10^6
2×10^6
1×10^6
0
***
BM
Spleen
(e)
Reticulocytes
cells / tissue
4×10^7
3×10^7
2×10^7
1×10^7
0
BM
Spleen
Saline
CSF1-Fc D14

Supp. Fig. 8

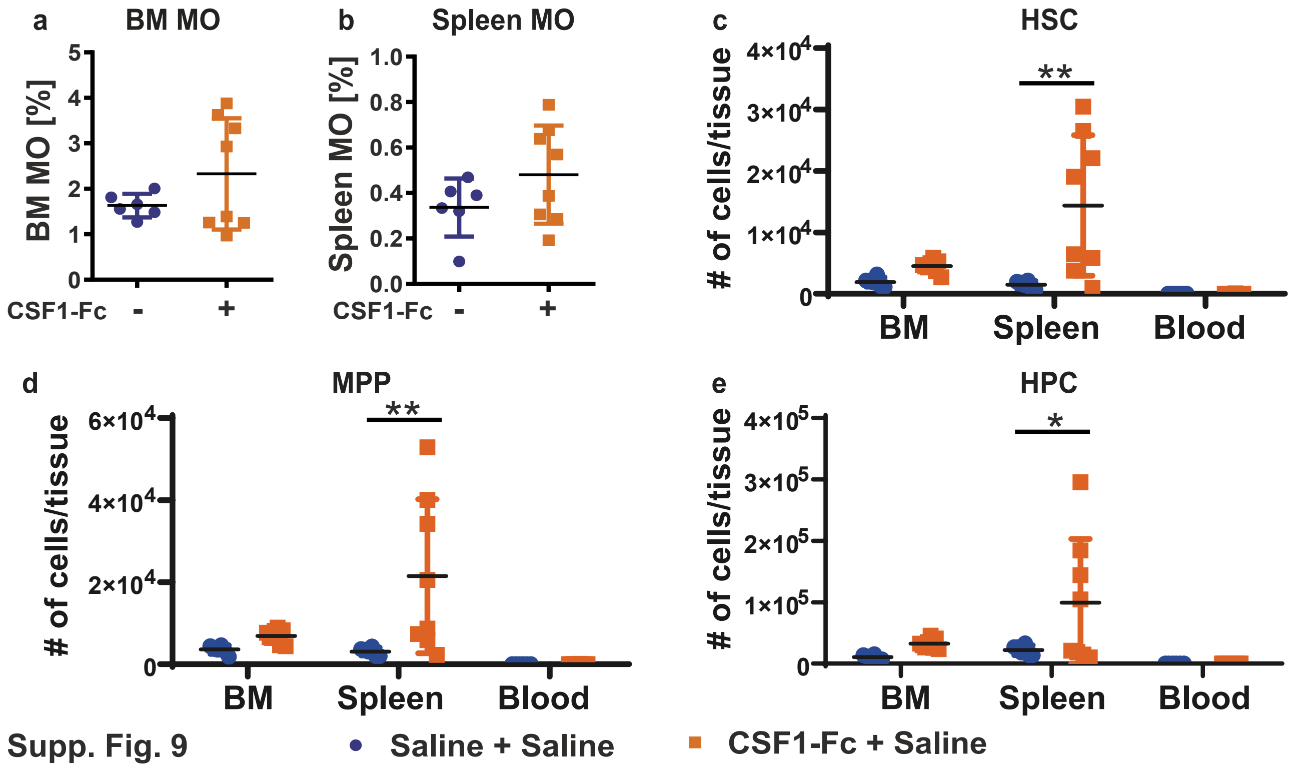
a
BM MO
BM MO [%]
CSF1-Fc
-
+
b
Spleen MO
Spleen MO [%]
CSF1-Fc
-
+
c
HSC
# of cells/tissue
**
BM
Spleen
Blood
d
MPP
# of cells/tissue
**
BM
Spleen
Blood
e
HPC
# of cells/tissue
*
BM
Spleen
Blood
Saline + Saline
CSF1-Fc + Saline

Supp. Fig. 9

Pre-gated on live, total CD45+ lymphocytes:

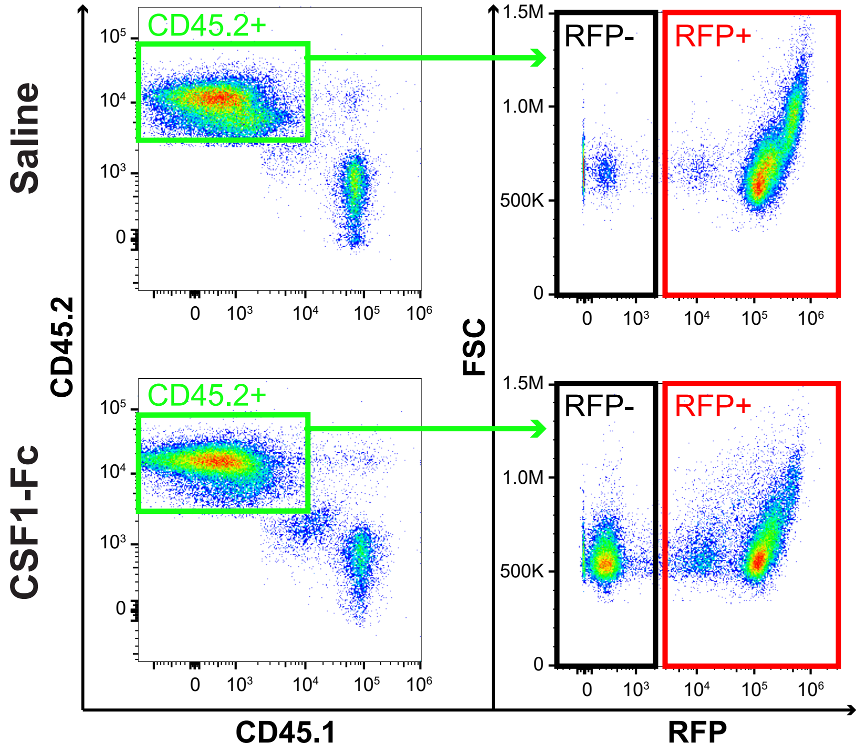

Supp. Fig. 10

Supplementary Table 1. Flow cytometry antibody details.

Panel/Tissues	Specificity	Flourochrome	Clone	Supplier	Catalogue Number
**Myeloid panel**	Ly6G	BV785	1A8	Biolegend®	127645
**All tissues**	CD115	BV605	AFS98	Biolegend®	135517
	CD11b	BV510	M1/70	Biolegend®	101245
	VCAM-1	BV421	51-10C9	BD Biosciences	744309
	CD169	PE-Cy7	3D6.112	Biolegend®	142411
	F4/80	AF647	BM8	Biolegend®	123121
**Lymphocyte Panel**	IgD	BUV395	11-26c.2a	BD Biosciences	564274
**All tissues**	CD93	BV650	AA4.1	BD Biosciences	563807
	IgM	BV605	RMM-1	Biolegend®	406523
	CD11b	BV510	M1/70	Biolegend®	101245
	CD4	PB	GK1.5	Biolegend®	100427
	CD3	FITC	17A2	Biolegend®	100203
	CD19	PerCP-CY5.5	1D3/CD19	Biolegend®	152405
	CD43	PE	S11	Biolegend®	143205
	CD8a	PE-Cy7	53-6.7	Biolegend®	100721
	CD23	AF700	B3B4	Biolegend®	101631
	CD45R(B220)	APC-Cy7	RA3-6B2	Biolegend®	103223
**LSK Panel**	CD3	FITC	17A2	Biolegend®	100203
**All tissues**	CD5	FITC	53-7.3	Biolegend®	100605
	CD11b	FITC	M1/70	Biolegend®	101205
	CD45R(B220)	FITC	RA3-6B2	BD Biosciences	553087
	GR-1	FITC	RB6-8C5	Biolegend®	108405
	TER119	FITC	TER-119	Biolegend®	116205

Panel/Tissues	Specificity	Flourochrome	Clone	Supplier	Catalogue Number
	CD150	BV650	TC15-12F12.2	Biolegend®	115931
	CD48	PB	HM48-1	Biolegend®	103417
	Sca-1	Pe-Cy7	E13-161.7	Biolegend®	122513
	cKit	APC	2B8	Biolegend®	105811
**Myeloid and Lymphoid Progenitors Panel**	IL-7Ra	BV785	A7R34	Biolegend®	135037
	Sca-1	BV510	D7	Biolegend®	108129
**All tissues**	CD3	PB	17A2	Biolegend®	100213
	CD5	PB	53-7.3	Biolegend®	100641
	CD11b	PB	M1/70	Biolegend®	101223
	CD45R(B220)	PB	RA3-6B2	Biolegend®	103230
	GR-1	PB	RB6-8C5	Biolegend®	108429
	TER119	PB	TER-119	Biolegend®	116231
	CD16/32	PE	93	Biolegend®	101301
	CD34	e660	RAM34	Thermo Fisher Scientific	50-0341-82
	cKit	APC-Cy7	2B8		105825
**Erythroblast Lineage Panel**	Hoechst 33342	N/A	N/A	Thermo Fisher Scientific	H3570
	CD71	PE	RI7217	Biolegend®	113807
	TER119	PE-Cy7	TER-119	Biolegend®	116221
	CD45	APC	30-F11	Biolegend®	103111
**Viability Dye**	7-Aminoactinomycin D (7AAD)	N/A	N/A	Thermo Fisher Scientific	A1310

Supplementary Table 2. Imaging flow cytometry antibody details

Specificity	Flourochrome	Clone	Supplier	Catalogue Number	Display Properties
CD3	Biotin	145-2C11	Biolegend®	100304	N/A
CD5	Biotin	53-7.3	Biolegend®	100604	N/A
CD11b	Biotin	M1/70	Biolegend®	101204	N/A
CD45R (B220)	Biotin	RA3-6B2	Biolegend®	103204	N/A
GR-1	Biotin	RB6-8C5	Biolegend®	108404	N/A
TER119	Biotin	TER-119	Biolegend®	116223	N/A
Streptavidin	APC-Cy7	N/A	Biolegend®	405208	
CD150	PE	TC15-12F12.2	Biolegend®	115904	35-180
CD48	PerCP-Cy5.5.	HM48-1	Biolegend®	103422	35-150
Sca-1	BV510	D7	Biolegend®	108129	35-450
cKit (CD117)	APC	2B8	Biolegend®	105812	30-150
CD115	BV605	AFS98	Biolegend®	135517	42-150
Csf1r-EGFP	GFP	N/A	N/A	N/A	30-500
